# Recycling of modified H2A-H2B provides short-term memory of chromatin states

**DOI:** 10.1016/j.cell.2023.01.007

**Published:** 2023-03-02

**Authors:** Valentin Flury, Nazaret Reverón-Gómez, Nicolas Alcaraz, Kathleen R. Stewart-Morgan, Alice Wenger, Robert J. Klose, Anja Groth

**Affiliations:** 1 Novo Nordisk Foundation Center for Protein Research, University of Copenhagen, 2200 Copenhagen, Denmark; 2 Biotech Research and Innovation Centre, University of Copenhagen, 2200 Copenhagen, Denmark; 3 Department of Biochemistry, University of Oxford, Oxford OX1 3QU, UK

**Keywords:** chromatin, DNA replication, histone recycling, H2A, H2B, H2A.Z, ubiquitination, histone PTM cross talk, polycomb, post-translational modifications

## Abstract

Chromatin landscapes are disrupted during DNA replication and must be restored faithfully to maintain genome regulation and cell identity. The histone H3-H4 modification landscape is restored by parental histone recycling and modification of new histones. How DNA replication impacts on histone H2A-H2B is currently unknown. Here, we measure H2A-H2B modifications and H2A.Z during DNA replication and across the cell cycle using quantitative genomics. We show that H2AK119ub1, H2BK120ub1, and H2A.Z are recycled accurately during DNA replication. Modified H2A-H2B are segregated symmetrically to daughter strands via POLA1 on the lagging strand, but independent of H3-H4 recycling. Post-replication, H2A-H2B modification and variant landscapes are quickly restored, and H2AK119ub1 guides accurate restoration of H3K27me3. This work reveals epigenetic transmission of parental H2A-H2B during DNA replication and identifies cross talk between H3-H4 and H2A-H2B modifications in epigenome propagation. We propose that rapid short-term memory of recycled H2A-H2B modifications facilitates restoration of stable H3-H4 chromatin states.

## Highlights

• Histone H2A-H2B are recycled during DNA replication independent of H3-H4
• H2A.Z and H2BK120ub1 mark nascent chromatin prior to transcription re-start
• H2A-H2B modification and variant landscapes are restored rapidly after replication
• H2AK119ub1 facilitates accurate and timely restoration of H3K27me3 post-replication

## Graphical abstract

**Figure:**
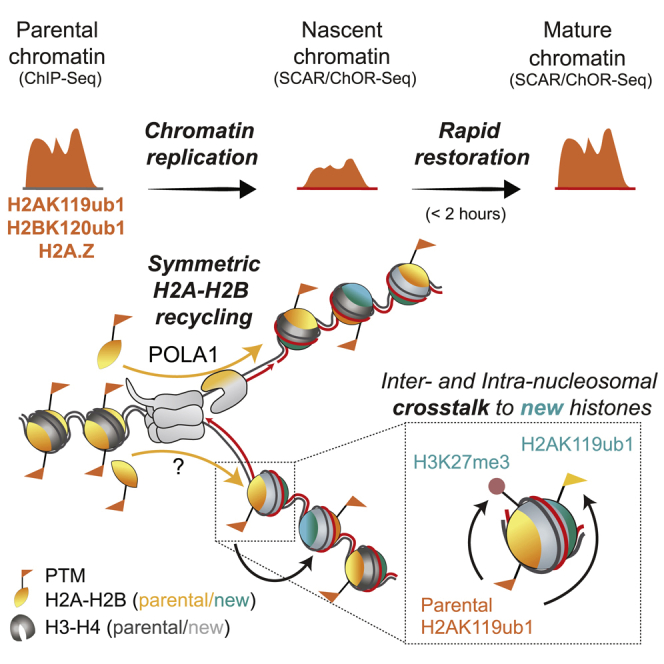

## Teaser

Accurate recycling of histones during DNA replication is crucial for restoring chromatin landscape and cell identity. A new study provides mechanistic insights into the elusive replication-dependent recycling of H2A-H2B and the associated histone modifications.

## Introduction

Regulation of gene expression is a highly complex, multi-layered process that governs cell identity. A major component in this regulation is chromatin, which exists in accessible and inaccessible states and orchestrates access to underlying genes. Establishment and maintenance of specific chromatin states is critical to ensure cell-type-specific gene expression programs.^1^ DNA replication is a constant threat to the maintenance of specialized chromatin landscapes, as the replication machinery disrupts chromatin during each round of cell division.^2^^,^^3^ How chromatin states are restored and propagated across cell division remains poorly understood.

A fundamental component of chromatin is the nucleosome, a histone octamer composed of a central H3-H4 tetramer and two flanking H2A-H2B dimers that packages 146 base pairs of DNA. Histones are decorated with a large variety of locus-specific post-translational modifications (PTMs).^4^ These PTMs influence DNA accessibility and gene expression by either directly affecting nucleosome stability and compaction or by recruiting a plethora of PTM reader proteins.^4^^,^^5^ Histone PTMs have long been considered an attractive source of epigenetic information, and histones H3-H4 are now established as a central unit for transferring epigenetic memory during DNA replication.^3^^,^^6^ Whether the same is true for H2A-H2B remains unknown.

Pioneering work in the 1980s revealed that old parental histones are recycled to the daughter strands during DNA replication and that old H3-H4 are maintained as tetramers and do not mix with new H3-H4 histones.^7^^,^^8^^,^^9^^,^^10^ However, old and new H2A-H2B dimers are found with both old and new H3-H4 tetramers and mixed in the same nucleosome, indicating that parental histone octamers are disassembled into H3-H4 tetramers and H2A-H2B dimers at the replication fork. Post-replication, the bulk of recycled H3-H4 tetramers largely remain at the site of incorporation and undergo limited exchange.^8^^,^^11^^,^^12^ In contrast, H2A-H2B undergo extensive replication independent exchange, partly due to eviction during transcription.^11^^,^^12^^,^^13^ Because of these dynamic properties, H2A-H2B behavior during replication could not be addressed by bulk metabolic/fluorescence labeling approaches.^8^^,^^11^ Hence, further characterization of histone recycling during DNA replication almost exclusively focused on H3-H4. Given that transmission to daughter DNA strands during replication is a pre-requisite for modified histones to propagate chromatin states across cell division, the contribution of H2A-H2B modifications to epigenetic cell memory remains debated.

Old and new histones H3-H4 are handled separately during DNA replication and differ extensively in their PTMs. New histones H3-H4 are hyperacetylated and generally lack di- and tri-methylation.^14^^,^^15^ Parental histones H3-H4 are recycled with their modifications in a highly accurate manner that maintains positional information in the histone PTM landscape.^16^^,^^17^ However, PTM levels are diluted 2-fold by the incorporation of new histones.^18^ Post-replication, new histone H3-H4 are modified to restore the chromatin landscape with locus- and modification-specific restoration kinetics.^16^^,^^18^ This process can be slow, as exemplified by H3 lysine 27 tri-methylation (H3K27me3) that is restored to pre-replication levels in G1 of the next cell cycle. Given that H3K27me3 is a key component of Polycomb-repressed heterochromatin,^19^ it remains unclear how repression is maintained in post-replicative chromatin.

Regulatory PTMs are also found on histones H2A and H2B, such as ubiquitination of H2A lysine 119 (H2AK119ub1) and H2B lysine 120 (H2BK120ub1). H2AK119ub1 forms repressive chromatin landscapes orchestrated by Polycomb-repressive complex 1 (PRC1) containing the ubiquitin ligases RING1A/B. H2AK119ub1 is involved in extensive cross talk with H3K27me3 and its writer Polycomb-repressive complex 2 (PRC2).^19^^,^^20^ H2BK120ub1 is a hallmark of transcribed chromatin where it promotes deposition of H3K4me3 and H3K79me3 over gene bodies.^21^ Besides H2A/H2B modifications, the histone variants H2A.Z, H2A.X, and macroH2A also have regulatory functions in transcription, DNA repair, and development.^22^ Despite being core nucleosome components and central hubs for the histone PTM landscape, it remains unknown how replication impacts on H2A-H2B and their variants.

Recent development of sequencing-based techniques, such as chromatin occupancy after replication (ChOR-seq), sister chromatid after replication (SCAR-seq), eSPAN, and others, has allowed interrogation of any chromatin component during replication in a time-sensitive and locus-specific manner.^16^^,^^23^^,^^24^ Most strikingly, the integration of these techniques with replication fork orientation has demonstrated that histones H3-H4 are recycled symmetrically to both daughter strands genome-wide.^23^^,^^24^ Subsequently, several replication factors with chaperone activity, including MCM2, POLE3/4, and POLA1, have been shown to recycle H3-H4 to either the leading or the lagging strand.^23^^,^^24^^,^^25^^,^^26^

To shed light on the fate of parental H2A-H2B during DNA replication, we took advantage of ChOR- and SCAR-seq to track occupancy of H2AK119ub1, H2BK120ub1, and H2A.Z on newly replicated DNA in mouse embryonic stem cells (mESCs). This revealed that H2A-H2B are recycled during DNA replication with similar efficiency and accuracy as H3-H4. H2A-H2B recycling is symmetric and operates through a distinct mechanism independent H3-H4 recycling, involving the lagging strand polymerase POLA1. We show that H2A-H2B landscapes are restored rapidly post-replication, in a time frame comparable to transcription re-start.^27^ Post-replication, H2AK119ub1 guides timely and accurate restoration of H3K27me3, attributing an important role to H2A-H2B recycling in propagating the epigenome. Connecting these findings with the dynamic nature of H2A-H2B modifications, we propose that short-term memory provided by H2A-H2B during DNA replication guides the maintenance of H3-H4 based long-term memory.

## Results

### H2A-H2B modifications in nascent chromatin mirror parental chromatin states

**Figure 1:**
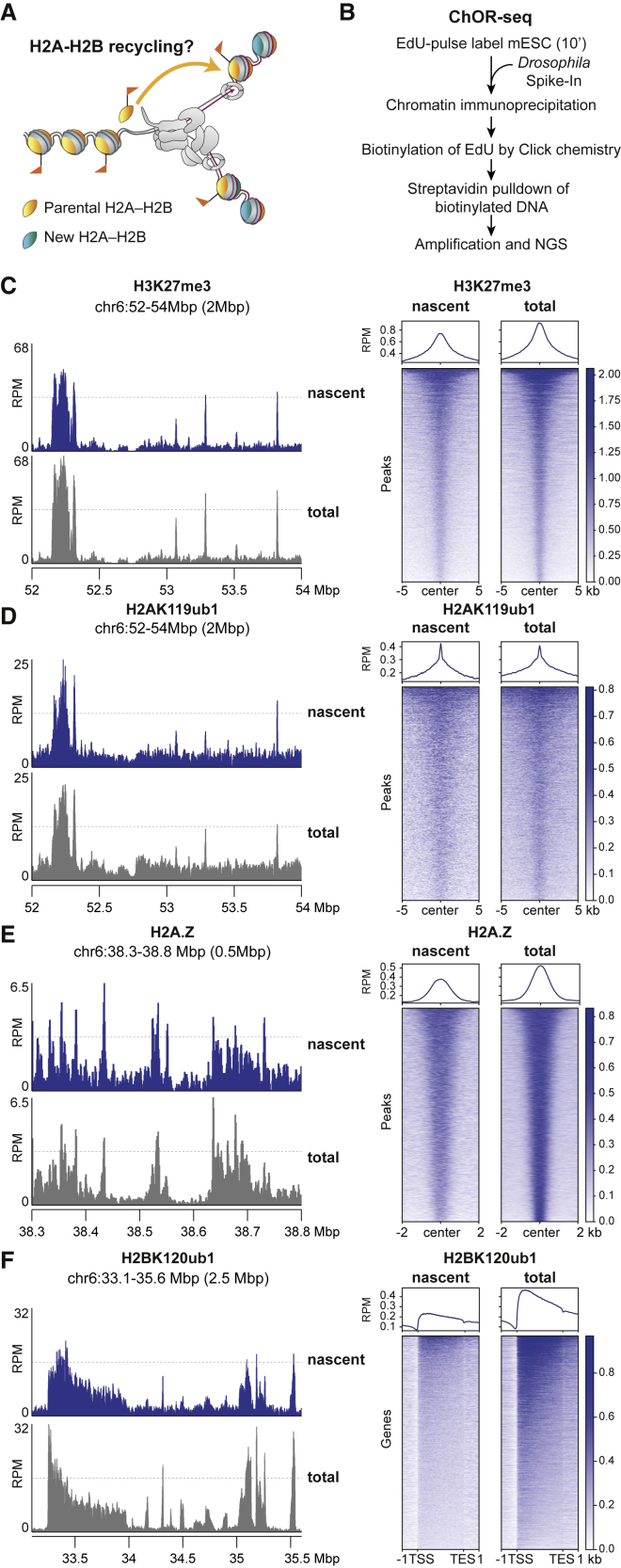
H2A-H2B modifications in nascent chromatin mirror parental chromatin states (A) Scheme outlining the question addressed in this study. Do recycling of modified H2A-H2B contribute to transmission of chromatin states during replication? Flags represent post-translational modifications. (B) Overview of the ChOR-seq workflow. (C–F) (Left) Nascent chromatin (ChOR-seq, blue) and total chromatin (ChIP-seq, gray) occupancy of H3K27me3, H2AK119ub1, H2A.Z, and H2BK120ub1 (C–E). (Right) Heatmap and average profile across peaks of H3K27me3, H2AK119ub1, and H2A.Z (F). (Right) Metagene analysis across H2BK120ub1-decorated genes. TSS: transcription start site; TES: transcription end site. Signal is sorted according to total ChIP-seq intensity and quantified with reads per million (RPMs). Data are represented as average of two replicates. See also Figure S1.

**Figure S1:**
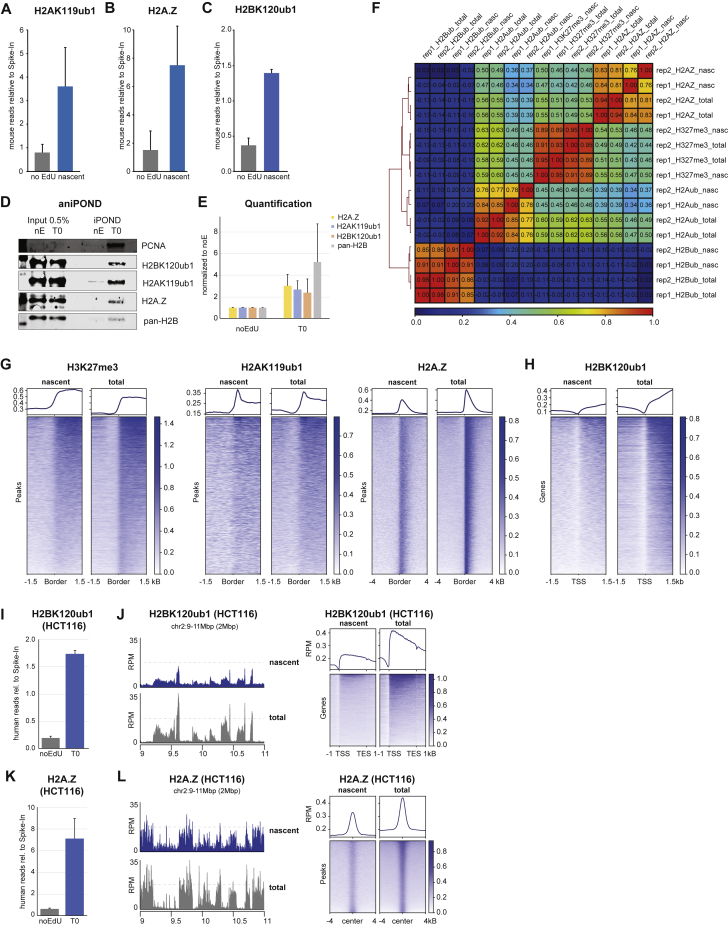
H2A-H2B modifications in nascent chromatin mirror parental chromatin states, related to Figure 1 (A–C) Barplot of mapped reads (mouse) relative to spike-in reads (*Drosophila*) in noEdU or nascent samples for H2AK119ub1 (A, n = 4), H2A.Z (B, n = 2), and H2BK120ub1 (C, n = 2). (D) Western blot analysis of aniPOND signal with indicated antibodies. A representative image is shown. nE: no EdU control. (E) Quantification of aniPOND signal of nascent (T0) relative to no EdU samples for the indicated antibodies. Average of three replicates. (F) Pearson correlation plot of replicates and conditions of the marks examined. RPM-normalized values were used to probe correlation. (G) Heatmap and average profile across peak boundaries (H3K27me3, H2AK119ub1, and H2A.Z, left to right). RPM scale. (H) Heatmap and average profile across TSS for H2BK120ub1-decorated genes. RPM scale. (I and K) Barplot of mapped reads (human) relative to spike-in reads (*Drosophila*) in noEdU or nascent samples for H2A.Z and H2BK120ub1. (J and L) (Left) Nascent chromatin (ChOR-seq, blue) and total chromatin (ChIP-seq, gray) occupancy of H2BK120ub1 and H2A.Z. (Right) Metagene analysis across H2BK120ub1-decorated genes (J) and heatmap and average profile across peaks of H2A.Z, (L) RPM scale. (G–L) Data are represented as average of two replicates.

To gain insights into how H2A-H2B modifications localize after DNA replication, we profiled the occupancy of H2AK119ub1, H2BK120ub1, and H2A.Z immediately after DNA replication (Figure 1A). To obtain high temporal resolution, we performed quantitative (q)ChOR-seq, using 5-ethynyl-2′-deoxyuridine (EdU)-labeled *Drosophila* S2 chromatin as a spike-in reference: asynchronous mESCs were pulse labeled 10 min with EdU, followed by chromatin immunoprecipitation (ChIP) and isolation of EdU-labeled DNA for next-generation sequencing^16^^,^^23^ (Figure 1B). Alongside qChOR-seq analysis of nascent newly replicated chromatin, we sequenced the total ChIP’ed material to obtain interphase genome-wide chromatin occupancy by standard ChIP-seq. While ChIP-seq and ChOR-seq signal intensity are not directly comparable as ChOR-seq involves an additional purification step of EdU-labeled DNA that changes signal-to-noise ratios,^16^ both occupancy patterns and relative peak intensities can be compared between nascent and total chromatin. We included H3K27me3 as a reference modification known to be recycled accurately during DNA replication.^16^^,^^18^ Importantly, no EdU control qChOR-seq samples showed very low read numbers across all experiments (Figures S1A–S1C), confirming that H2AK119ub1, H2BK120ub1, and H2A.Z qChOR-seq represent occupancy specifically in EdU-labeled nascent chromatin. Moreover, the presence of H2AK119ub1, H2BK120ub1, and H2A.Z in nascent chromatin was recapitulated by native iPOND (Figures S1D and S1E). Comparison of the qChOR-seq and ChIP-seq signals for H2A.Z and H2AK119ub1 revealed that the occupancy pattern on nascent chromatin only 10 min after replication fork passage mirrored the genome-wide occupancy in total chromatin with respect to signal position and relative signal intensity among peaks (Figures 1C–1E, S1F, and S1G), as previously described for H3K27me3.^16^ H2BK120ub1 was also detected in nascent chromatin localizing across gene bodies similar to H2BK120ub1 in total chromatin, but with a reduced occupancy in 5′ regions as compared with gene bodies (Figures 1F and S1H). This shifted occupancy pattern might reflect higher H2BK120ub1 dynamics in the 5′ regions of genes.^28^ Importantly, H2A.Z and H2BK120ub1 showed a similar occupancy pattern in nascent chromatin also in human HCT116 cells (Figures S1I–S1L). Collectively, this demonstrates that modifications on H2A-H2B and the H2A.Z variant are present in nascent chromatin largely mirroring total chromatin occupancy, similar to H3-H4 methylation.^16^

### Parental H2AK119ub1 and H2BK120ub1 are recycled during DNA replication

**Figure 2:**
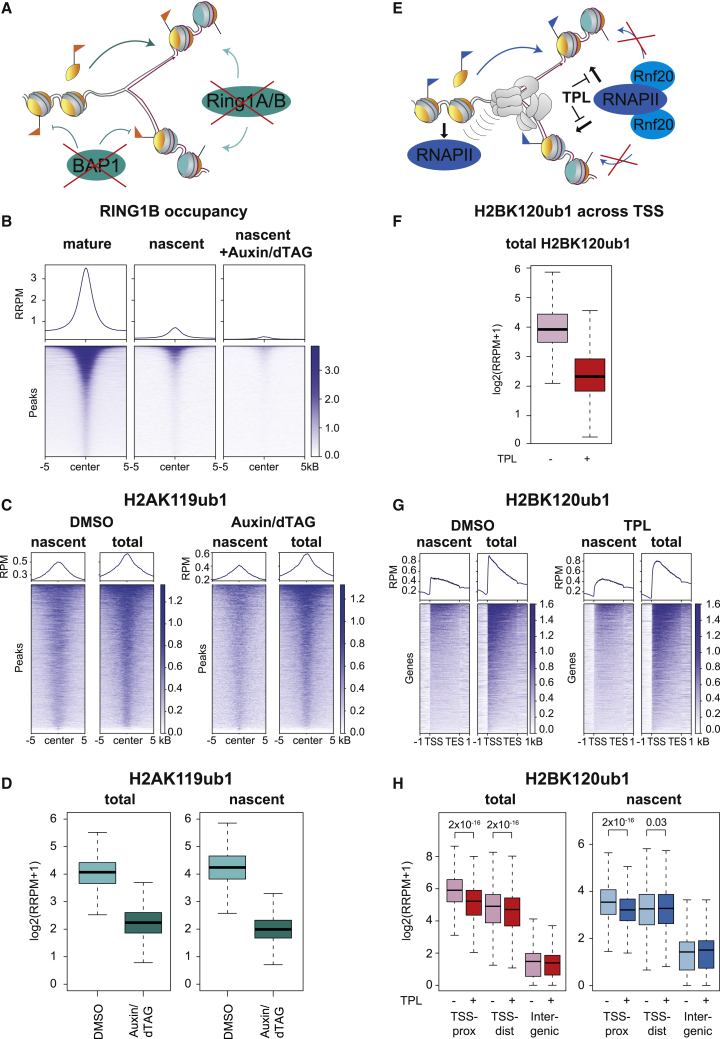
Parental H2AK119ub1 and H2BK120ub1 are recycled during DNA replication (A) Strategy to prevent *de novo* H2AK119ub1 (orange flag) by depletion of RING1A/B and co-depletion of BAP1 to prolong the half-life of H2AK119ub1. (B) Heatmap and average profile of RING1B occupancy across RING1B peaks on mature and nascent chromatin with or without RING1B/BAP1 depletion. Signal is quantified with reference-adjusted reads per million using exogenous spike-in chromatin (RRPM). (C) Heatmap and average profile across H2AK119ub1 peaks of nascent and total H2AK119ub1 occupancy in untreated (left) or dTAG/Auxin-treated cells (right). Signal is sorted according to total ChIP-seq intensity. RPM scale. (D) Signal across H2AK119ub1 peaks in total or nascent chromatin. Log_2_(RRPM + 1) scale. Black line, median; dashed lines, 1.5× interquartile range. (E) Strategy to prevent *de novo* H2BK120ub1 (blue flag) by inhibiting transcription re-start using triptolide (TPL) with simultaneous EdU labeling (10 min). Thick arrows visualize eviction of RNAPII prior to replication and recruitment post-replication. (F) Signal of total H2BK120ub1 in 1 kb windows overlapping the TSS upon DMSO or TPL treatment. Log_2_(RRPM + 1) scale. Black line, median; dashed lines, 1.5× interquartile range. (G) Heatmap and average profile of nascent and total H2BK120ub1 occupancy across H2BK120ub1-decorated genes (>50 kB) in untreated (left) or treated cells. Signal is sorted according to total ChIP-seq intensity. RPM scale. (H) Signal of H2BK120ub1 across genic (TSS-prox: 0–10 kb from TSS, TSS-dist: >50 kb from TSS) or intergenic regions in total or nascent chromatin. Log_2_(RRPM + 1) scale. Black line, median; dashed lines, 1.5× interquartile range. Statistics by unpaired Wilcoxon test. Data are represented as average of three (C and D), or two (B and F–H) replicates. See also Figure S2.

**Figure S2:**
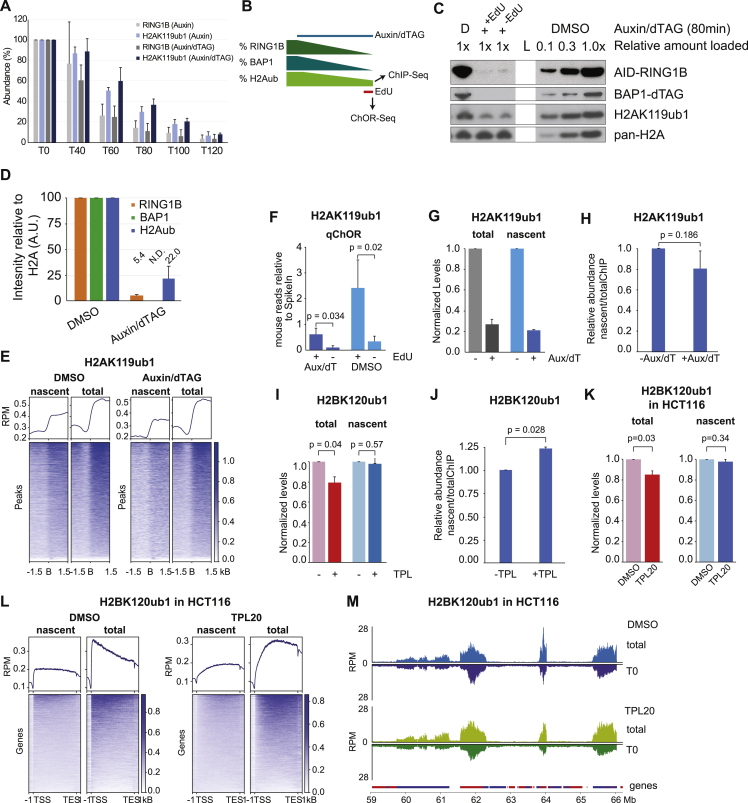
Parental H2AK119ub1 and H2BK120ub1 are recycled during DNA replication, related to Figure 2 (A) BAP1/RING1B signal over time analyzed by western blot in single or double depletion time courses. Average of three independent replicates. Western blot was quantified using ImageJ. (B) Experimental setup. Depletion of BAP1 and RING1B was initiated by addition of Auxin/dTAG until completion, and thereafter, EdU was added to analyze nascent chromatin occupancy of residual H2AK119ub1. (C) Western blot Analysis with DMSO-treated dilution curve as reference. Note that the ladder (L; lane 4) has been removed from the Image. “D” refers to DMSO-treated controls, while “+” refers to Auxin/dTAG treatment for 80 min. (D) Quantification of (C), average of three independent replicates. (E) Heatmap and average profile of nascent and total H2AK119ub1 occupancy across H2AK119ub1 peak boundaries. RPM scale. (F) Barplot of mapped reads (mouse) relative to spike-in reads (*Drosophila*) in noEdU or nascent samples for H2AK119ub1 in cells treated with DMSO or Auxin/dTAG. (G) Barplot of mapped reads (mouse) relative to spike-in reads (*Drosophila*) for H2AK119ub1 in cells treated with DMSO or Auxin/dTAG. Normalized to input. (H) Barplot of ChOR-intensity normalized to ChIP-intensity in Auxin/dTAG-treated cells. Normalized to DMSO condition. (I) Barplot of mapped reads (mouse) relative to spike-in reads (*Drosophila*) for H2BK120ub1 in mES cells treated with DMSO or TPL (Triptolide). Normalized to input. (J) Barplot of ChOR-intensity normalized to ChIP-intensity in TPL-treated cells. Normalized to DMSO condition. (K) Barplot of mapped reads (human) relative to spike-in reads (*Drosophila*) for H2BK120ub1 in HCT116 cells treated with DMSO or TPL20 (20 min TPL treatment). Normalized to input. (L) Heatmap and average profile of nascent and total H2BK120ub1 occupancy across H2BK120ub1-decorated genes (>50 kB) in DMSO (left) or TPL20-treated (right) HCT116 cells. Signal is sorted according to total ChIP-seq intensity. RPM scale. (M) Occupancy tracks for H2BK120ub1 in DMSO (top) or TPL20-treated (bottom) cells. RPM scale. Data are represented as average of three (C–H) and two (I–N) replicates. Statistics by Student t test (two-tailed, unpaired).

Our qChOR-seq analysis argues H2A-H2B could be recycled during DNA replication. However, H2AK119ub1 and H2BK120ub1 are dynamic modifications,^29^^,^^30^ which might be imposed on newly assembled chromatin already during the 10 min EdU labeling step. To exclude this possibility, we prevented *de novo* deposition by two orthogonal approaches. First, we rapidly depleted writer enzymes for H2AK119ub1 using RING1B fused to an Auxin-inducible degron (AID) in a RING1A^−/−^ background (Figure 2A).^31^ H2AK119ub1 was turned over rapidly upon RING1A/B depletion (Figure S2A)^31^^,^^32^ and we thus sought to prolong the half-life of H2AK119ub1 by removing the deubiquitinase BAP1 using a dTAG degron.^33^^,^^34^^,^^35^ Using this system, we identified a time window around 80 min of Auxin/dTAG treatment were RING1B was depleted by 95%, while around 25% of H2AK119ub1 remained on chromatin as indicated by western blotting and quantitative ChIP-seq (Figures S2B–S2D). qChOR-seq for RING1B confirmed that RING1B is very low abundant on nascent chromatin compared with mature chromatin (2 h chase) and virtually undetectable upon dTAG/Auxin treatment (Figure 2B). In this setting, H2AK119ub1 should only be detected on nascent chromatin if transmitted by recycling of H2A-H2B, and we therefore performed qChOR-seq for H2AK119ub1. In the absence of *de novo* deposition, the occupancy of nascent H2AK119ub1 remained significantly above background (noEdU control) and the pattern again recapitulated that of total H2AK119ub1 (Figures 2C, S2E, and S2F). Quantitatively, nascent H2AK119ub1 levels were reduced upon RING1B/BAP1 depletion, but to an extent comparable to the reduction in total H2AK119ub1 both assessing levels genome-wide and across peaks (Figures 2D, S2G, and S2H). This argues that recycling of modified H2A-H2B during DNA replication is mainly responsible for the H2AK119ub1 landscape on nascent chromatin.

H2BK120ub1 demarcation of genes is established during transcription by RNAPII-mediated recruitment of the RNF20-RNF40 ubiquitin ligase complex.^36^^,^^37^^,^^38^ However, nascent chromatin is inaccessible and transcriptionally silent for at least 10 min after fork passage in mESCs,^27^ arguing that transcription re-start is unlikely to explain H2BK120ub1 occupancy immediately after replication. Consistent with this, longer genes (>50 kilobases [kb]) requiring more than 10 min to be transcribed were marked by H2BK120ub1 in nascent chromatin (Figure 1F). However, as transcription re-start is heterogeneous,^27^ we used triptolide (TPL) (Figure 2E), a potent fast-acting transcription initiation inhibitor^39^^,^^40^ to clearly separate H2BK120ub1 recycling from *de novo* deposition. We added TPL during the EdU labeling step and used quantitative ChOR- and ChIP-seq to measure H2BK120ub1 abundance in nascent and total chromatin. As expected,^41^ TPL treatment significantly reduced H2BK120ub1 levels at transcription start sites (TSSs), consistent with a lack of initiation (Figure 2F). In total chromatin the loss was moderate, as TPL does not affect elongating RNAPII (Figure S2I). In the absence of transcription initiation post-replication, nascent H2BK120ub1 still mirrored total occupancy with a shift of signal toward the gene body (Figure 2G). We then quantified the effects of TPL inhibition on total and nascent chromatin at genic regions with varying distances to TSSs (Figure 2H). H2BK120ub1 abundance was affected more at TSS-proximal sites (<10 kb from TSS) as compared with more distal sites (>50 kb), while the background signal at intergenic sites remained largely unaltered. Importantly, H2BK120ub1 in nascent chromatin was less sensitive to TPL treatment as compared with total chromatin, with TSS-distal regions remaining essentially unaffected and TSS-proximal regions dropping less than total chromatin (Figures 2H and S2J). Similarly, 20 min TPL treatment (added 10 min prior to EdU) of human HCT116 cells did not affect the relative abundance of H2BK120ub1 on nascent chromatin as compared with total chromatin, and nascent H2BK120ub1 still accurately recapitulated the occupancy of total H2BK120ub1 (Figures S2K–S2M). Again, the high TSS-proximal H2BK120ub1 levels were most sensitive to TPL in total and nascent chromatin, likely reflecting inhibition of dynamic RNAPII turnover^42^ and lower H2BK120ub1 levels at these sites prior to replication fork passage. Collectively, these results show that transcription re-start is not needed to establish the nascent H2BK120ub1 landscape and argues that H2BK120ub1 is recycled during DNA replication.

### H2A-H2B is recycled symmetrically during DNA replication independent of H3-H4

**Figure 3:**
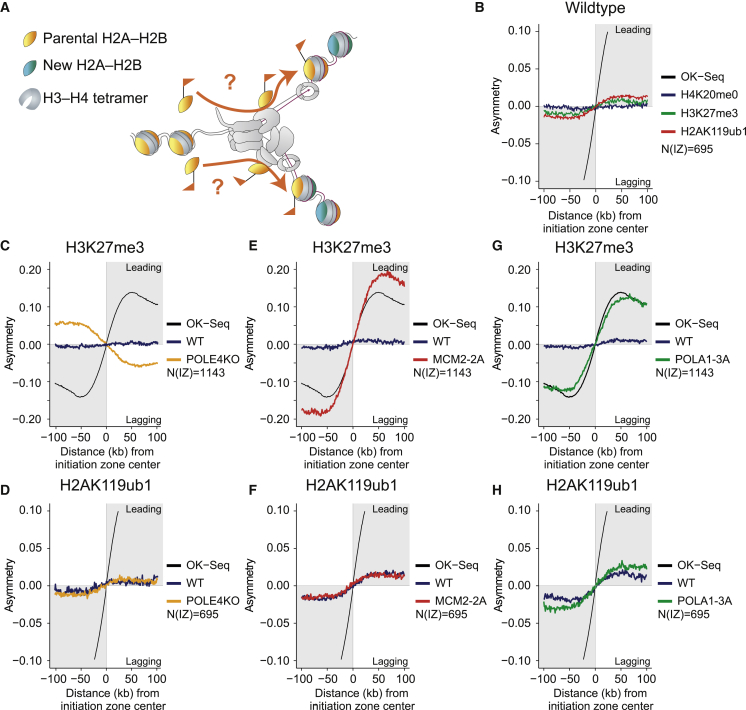
H2A-H2B is recycled symmetrically during DNA replication independent of H3-H4 (A) Illustration of the question addressed. Is H2A-H2B segregated symmetrically to both daughter strands? (B) Average SCAR-seq profile, showing replication fork directionality (RFD, measured by OK-seq), and asymmetry (measured as partition to leading and lagging strand), for the indicated histone PTMs across all replication initiation zones with a H2AK119ub1 peak. (C and D) Average SCAR-seq profile for H3K27me3 or H2AK119ub1 in WT or POLE4KO cells. (E and F) Average SCAR-seq profile for H3K27me3 or H2AK119ub1 in WT or MCM2-2A cells. (G and H) Average SCAR-seq profile for H3K27me3 or H2AK119ub1 in WT or POLA1-3A cells. (B), (D), (F), and (H): Note the change in scale to visualize smaller biases. Individual replicates shown in Figure S3. Data in (C), (E), and (G) are represented as average of n = 2–4 replicates. N(IZ): number of initiation zones analyzed (STAR Methods). See also Figure S3.

**Figure S3:**
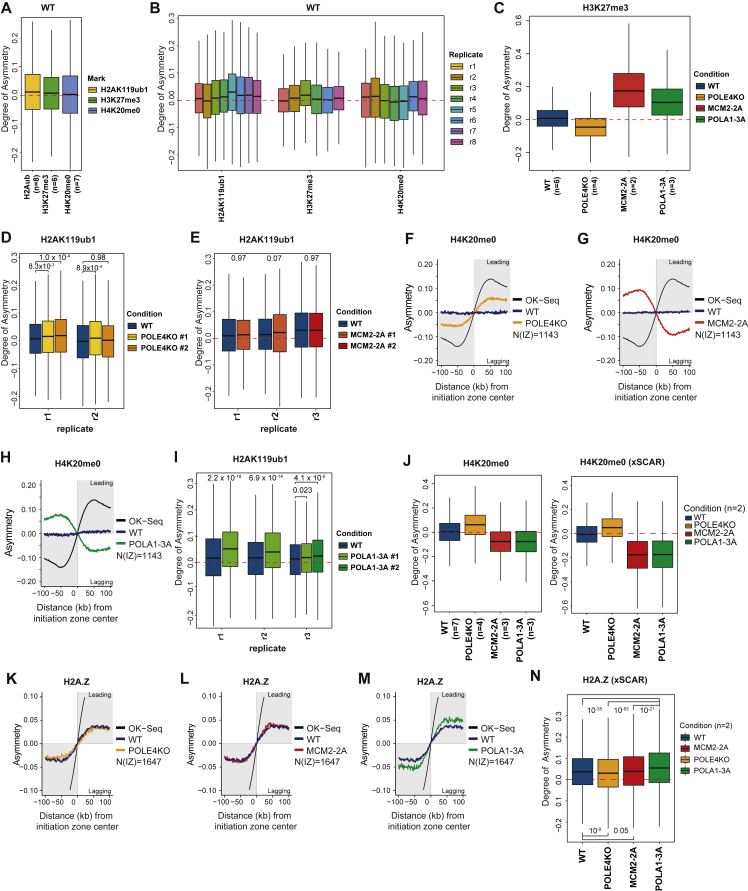
H2A-H2B is recycled symmetrically during DNA replication independent of H3-H4, related to Figure 3 (A) Asymmetry plot for H2AK119ub1, H3K27me3, and H4K20me0 across H2AK119ub1 peaks (n = 6–8). Positive values indicate leading strand bias. Negative values indicate bias toward lagging strand. (B) Asymmetry plot for H2AK119ub1, H3K27me3, and H4K20me0 across H2AK119ub1 peaks (n = 6–8). Individual replicates. (C) Asymmetry plot for H3K27me3 across called H3K27me3 peaks in different cell lines (n = 3–6). (D) Asymmetry plot for H2AK119ub1 across H2AK119ub1 peaks in POLE4KO cells, individual replicates (n = 2 for WT, n = 4 for 2 POLE4KO clones). (E) Asymmetry plot for H2AK119ub1 across H2AK119ub1 peaks in MCM2-2A cells, individual replicates (n = 3 for WT and 2 MCM2-2A clones). (F–H) Average SCAR-seq profile for H4K20me0 in WT and POLE4KO (F), MCM2-2A (G), or POLA1-3A (H) cells. (I) Asymmetry plot for H2AK119ub1 across H2AK119ub1 peaks in POLA1-3A cells, individual replicates (n = 3 for WT, n = 4 for 2 POLA1-3A clones). (J) Asymmetry plot for H4K20me0 in different cell lines (n = 3–4). (K–M) Average xSCAR-seq profile for H2A.Z in WT and POLE4KO (K), MCM2-2A (L), or POLA1-3A (M) cells (n = 2). (N) Asymmetry plot for H2A.Z in different cell lines (n = 2). (D, E, I, and N) Statistics by unpaired Wilcoxon test.

We next investigated whether H2A-H2B are recycled symmetrically to the two daughter strands similar to H3-H4 (Figure 3A). To determine the relative occupancy of recycled H2A-H2B on the two daughter DNA strands, we employed SCAR-seq, a technology that allows the measurement of histone PTM partition to sister chromatids.^43^ To assign SCAR-seq reads to either the leading or the lagging strand, we used Okazaki sequencing (OK-seq) to map replication fork directionality with respect to replication initiation zones, as previously described.^23^ To track parental H2A-H2B partitioning to leading and lagging strands we used H2AK119ub1 as a proxy and included H3K27me3 and H4K20me0 for comparison as markers of parental and newly synthesized H3-H4, respectively. This analysis revealed that H2AK119ub1 is recycled almost symmetrically to the two daughter strands, with a small leading strand bias similar to H3K27me3 (Figures 3B, S3A, and S3B). We next explored whether H2A-H2B recycling is mediated by the H3-H4 recycling pathways recently identified. POLE3/4 is a non-catalytic component of the DNA polymerase epsilon complex, required for recycling of parental H3-H4 to the leading strand.^24^^,^^26^ Deletion of POLE4 resulted in biased recycling of H3K27me3 toward the lagging strand, but the symmetric distribution of H2AK119ub1 was not altered (Figures 3C, 3D, S3C, and S3D). MCM2, part of the replicative helicase, recycles H3-H4 to the lagging strand via its histone-binding domain.^23^^,^^25^ Mutation of the MCM2 histone-binding domain (MCM2-2A) caused a strong asymmetry toward leading strand for H3K27me3, but once again, segregation of H2AK119ub1 remained symmetric (Figures 3E, 3F, S3C, and S3E). This argues that H2A-H2B are not recycled together with H3-H4, revealing the existence of a separate H2A-H2B recycling pathway. Old H2A-H2B can form nucleosomes with new H3-H4,^9^ but redeposition of parental H2A-H2B is also not linked to *de novo* deposition of H3-H4 as H4K20me0 showed a strong deposition bias opposite to that of H3K27me3 (Figures S3F and S3G).

Next, we focused on POLA1, the catalytic subunit of the DNA polymerase alpha complex acting on the lagging strand.^44^ POLA1 facilitates H3-H4 recycling to the lagging strand but can also bind histones H2A-H2B.^26^^,^^45^ Mutation of the POLA1 histone-binding domain (POLA1-3A) resulted in a clear bias of H3K27me3 toward the leading strand and an opposite bias of H4K20me0 to the lagging strand (Figures 3G, S3C, and S3H). We also identified a bias of H2AK119ub1 to the leading strand that was moderately, but consistently, enhanced in POLA1-3A compared with WT cells (Figures 3H and S3I). To further confirm these results, we extended our investigation to histone H2A.Z by performing xSCAR-seq (STAR Methods) with H4K20me0 as a control (Figure S3J). This recapitulated our data for H2AK119ub1, showing H2A.Z recycling independent of MCM2 and POLE4, while the leading strand bias was enhanced in POLA1-3A cells (Figures S3K–S3N). This reveals a role for POLA1 in H2A-H2B recycling to the lagging strand, suggesting that POLA1 serves as a landing platform not only for parental H3-H4 but also H2A-H2B.

### H2A-H2B modifications restore accurately and rapidly after DNA replication

**Figure 4:**
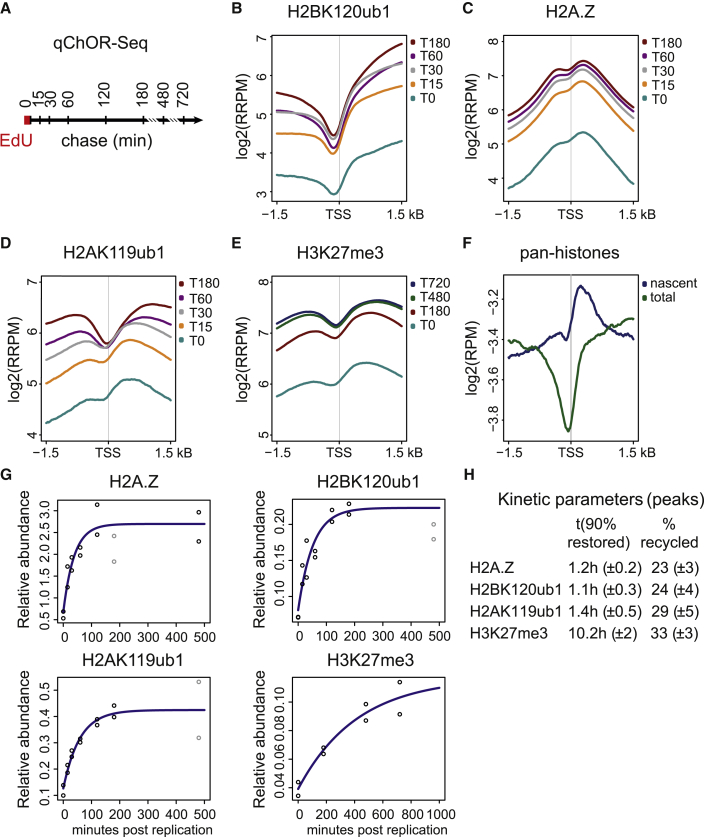
H2A-H2B marks restore accurately and rapidly after DNA replication (A) Experimental outline of the quantitative ChOR-seq time course. (B–E) Average profile of RRPM-normalized occupancy signal for H2BK120ub1, H2A.Z, H2AK119ub1, and H3K27me3 signal across 3 kb centered on the TSS. Only TSSs occupied by the respective mark were included. Log_2_ scale. (F) Average profile of RPM-normalized occupancy signal of nascent or total pan-histones (combined pan-H2A and pan-H3) across all TSSs (n = 30,025). Log_2_ scale. (G) Restoration curve for relative abundance of H2BK120ub, H2A.Z, H2AK119ub1, and H3K27me3 post-replication. Data points in gray were excluded from regression analysis (STAR Methods). (H) Kinetic parameters for investigated marks. t(90% restored): relative time (in hours) needed to restore 90% of the total signal. %recycled: estimated abundance at nascent chromatin (T0) across peaks. Data are represented as average of two replicates. See also Figure S4.

**Figure S4:**
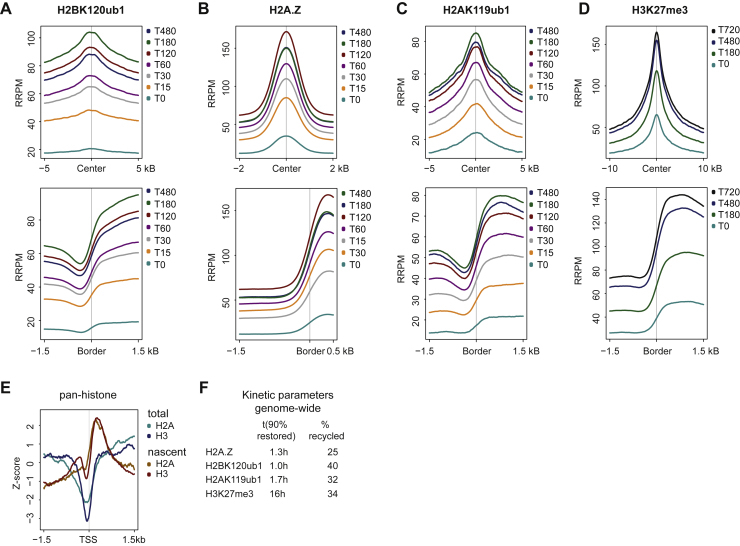
H2A-H2B marks restore accurately and rapidly after DNA replication, related to Figure 4 (A–D) Average profile of RRPM-normalized occupancy signal across peak centers (top) or boundaries (bottom) for H2BK120ub1, H2A.Z, H2AK119ub1, and H3K27me3. (E) Average profile of RPM-normalized occupancy signal of nascent or total pan-H2A or pan-H3 across all TSS, (n = 30,025). *Z* score normalized. (F) Kinetic parameters for investigated marks. t(90% restored): relative time (in hours) needed to restore 90% of the total signal. % recycled: estimated abundance at nascent chromatin (T0) across the entire genome. Considering all regions may not represent as accurate parameters as in contrast to Figure 4H. Data are represented as average of two replicates.

Next, we investigated how the H2A-H2B modification landscape restores over time with respect to both position and abundance by employing qChOR-seq time course analysis (Figure 4A). Peak boundaries and peak centers were clearly demarcated and maintained at all time points for all the modifications investigated, indicating accurate recycling and domain-based restoration for H2A and H2B modifications (Figures S4A–S4D). To gain further insights into recycling and restoration accuracy, we characterized histone PTM signals at TSSs—sites that are well annotated and contain a nucleosome-depleted region (NDR) just upstream of the TSS to facilitate transcription.^46^^,^^47^ Nascent and maturing H2BK120ub1 signal at TSSs were clearly restricted to regions downstream of the TSS, supporting accurate recycling (Figure 4B). H2A.Z, H2AK119ub1, and H3K27me3 also showed a clearly defined NDR that stayed constant over time, although with reduced demarcation in nascent chromatin (Figures 4C–4E). This is unexpected, given that DNA accessibility measured by MINCE-seq and repli-ATAC show NDRs are erased during DNA replication through the deposition of histones.^27^^,^^47^ Consistent with this, nascent ChOR-seq for pan-H2A/H3 confirmed that the NDR is indeed lost immediately after replication (Figures 4F and S4E). Since the loss of the NDR is not due to recycling of parental histones, it is likely connected to replication-coupled deposition of newly synthesized histones.

Next, we determined the restoration kinetics of modified H2A-H2B, focusing on the quantitative ChOR-seq signal localized to peaks identified in total ChIP-seq (Figure 4A). H2AK119ub1, H2BK120ub1, and H2A.Z all showed fast kinetics with full restoration in less than 3 h and prior to cell division (Figure 4G). The restoration measurements resembled a first-order reaction rate,^48^ where new histones get modified until a plateau phase is achieved where writing and erasing activities are balanced. Estimation of the restoration rates revealed that H2A.Z and H2BK120ub1 levels are restored (>90%) within 60–80 min and H2AK119ub1 levels within 2 h, in stark contrast to H3K27me3 that takes 10–12 h (almost a full cell cycle in mESCs) to restore (Figures 4H and S4F). The latter is consistent with previous work showing slow restoration of repressive modifications such as H3K27me3, H3K9me3, and H4K20me3 in mESCs and HeLa cells.^16^^,^^18^^,^^49^ H2AK119ub1 thus stands out as a repressive histone modification restored prior to cell division.

Fitting our restoration time course measurements to a first-order reaction also allowed an estimate of the initial levels of histone modifications/variant on nascent chromatin. The starting levels were in the range of 25%–35% for both H2A-H2B modifications, H2A.Z and H3K27me3 (Figures 4G and 4H). Although lower than 50% (see discussion), these number are consistent with the around 2-fold dilution of histone modifications during DNA replication due to deposition of new histones.^18^ In conclusion, our restoration analysis revealed that H2A-H2B modifications associated with both transcriptional activity and repression are accurately localized in nascent chromatin and steady-state levels are restored with fast kinetics prior to mitosis.

### H2A.Z and H2BK120ub1 restoration correlate with transcription

**Figure 5:**
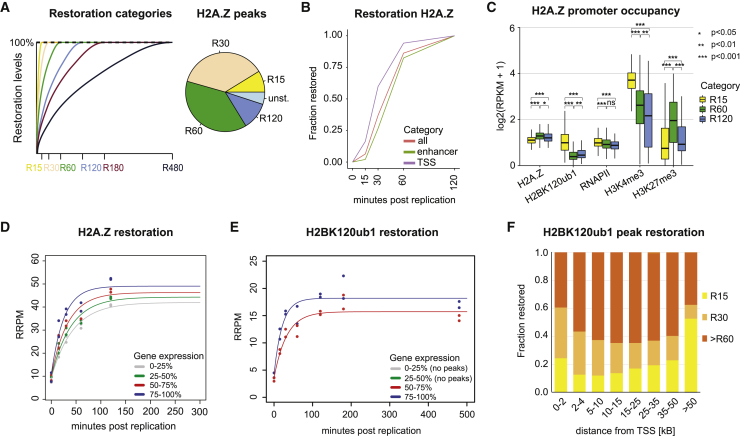
H2A.Z and H2BK120ub1 restoration correlate with transcription (A) Outline of restoration categories for H2A.Z peaks. Peaks are classified as restored once their signal show no further increase in all subsequent timepoints (for example, R60: restored at 60 min) or classified as unstable if their signal is decreasing in subsequent time points (STAR Methods). (B) Restoration kinetics of peaks overlapping enhancers or promoters (within 1 kb). (C) Promoter signature (within 1 kb from TSS) for the different restoration categories for H2A.Z. Log_2_(RPKM + 1) scale. Statistics by unpaired Wilcoxon test. ^∗^p < 0.05, ^∗∗^p < 0.01, ^∗∗∗^p < 0.001. H3K4me3 data from Sethi et al.,^51^ RNAPII-Ser5p data from Stewart-Morgan et al.^27^ (D) Restoration curve for H2A.Z peaks at promoters stratified according to gene expression level quartiles. (E) As in (D) but for H2BK120ub1 peaks. No peaks overlapped promoters at low- or not-expressed genes. (F) Relative restoration categories according to distance of H2BK120ub1 peaks to TSS. Data are represented as average of two replicates. See also Figure S5.

**Figure S5:**
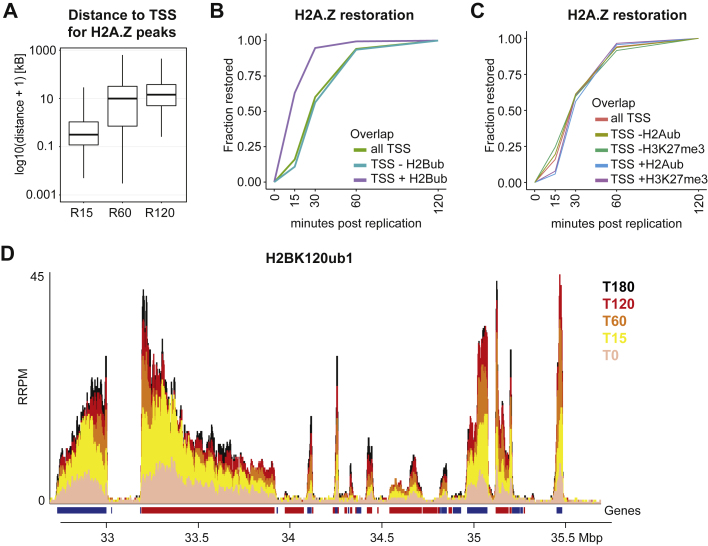
H2A.Z and H2BK120ub1 restoration correlate with transcription, related to Figure 5 (A) Box plot showing distance to TSS for H2A.Z peak restoration categories. Log_10_ scale. (B) Restoration lineplot for H2A.Z peaks mapping to promoters stratified according to overlap with H2BK120ub1 peaks. (C) Restoration lineplot for H2A.Z peaks mapping to promoters stratified according to overlap with H3K27me3 or H2AK119ub1 peaks. (D) Genomic occupancy of H2BK120ub1 across the time course. RRPM scale. Data are represented as average of two replicates.

Both H2A.Z and H2BK120ub1 have been linked to actively transcribed chromatin. To address how active chromatin landscapes are restored, we first classified peaks according to restoration time post-replication (STAR Methods). This revealed H2A.Z peaks that restore very quickly (R15) while others took more than 60 min to restore (Figure 5A). Peaks with decreasing H2A.Z levels across the time course were identified as unstable. Fast-restoring peaks localized closer to TSSs than medium (R60) and slowly (R120) restoring ones (Figure S5A), and H2A.Z at promoters restored faster than at enhancers (Figure 5B). Furthermore, fast-restoring H2A.Z loci showed high steady-state levels of H3K4me3 and H2BK120ub1 as well as increased RNAPII occupancy (Figures 5C and S5B). The same trend was also observed when classifying H2A.Z restoration kinetics according to gene expression, where highly expressed genes restore H2A.Z at their promoters faster than medium or lowly expressed genes (Figure 5D). Conversely, presence of H3K27me3 or H2AK119ub1 correlated with delayed restoration of H2A.Z (Figure S5C). Thus, H2A.Z restoration is accelerated in transcriptionally active chromatin, likely because of transcription re-start. This agrees with H3K4me3 acting upstream of and facilitating H2A.Z deposition.^50^ Considering that H2A.Z can regulate PRC2 recruitment,^50^ recycled H2A.Z may act as a placeholder to prevent gene silencing until transcription is restored.

Similar to H2A.Z, H2BK120ub1 peaks at promoters also restored faster on higher expressed genes (Figure 5E), suggesting that restoration of H2BK120ub1 is also driven by transcription re-start. Analysis of restoration across gene bodies is non-trivial due to variation of H2BK120ub1 signal according to gene length and structure (Figure S5D). However, classification of peaks according to their distance to TSS (STAR Methods) illustrated that fast-restoring peaks localized adjacent to the TSSs, and peaks at intermediate distance to the TSS restored slower (Figure 5F), as expected if restoration is driven by RNAPII progression. However, a substantial proportion of peaks localized distant to TSSs showed fast restoration (Figure 5F), which implies a restoration mechanism different from RNAPII progression from the TSS across the gene.

Collectively, H2BK120ub1 and H2A.Z landscapes are restored within 2 h after replication similar to chromatin accessibility and RNAPII occupancy,^27^ consistent with transcription re-initiation as a main driver.

### Variant PRC1 (vPRC1) sites show fastest H2AK119ub1 restoration

**Figure S6:**
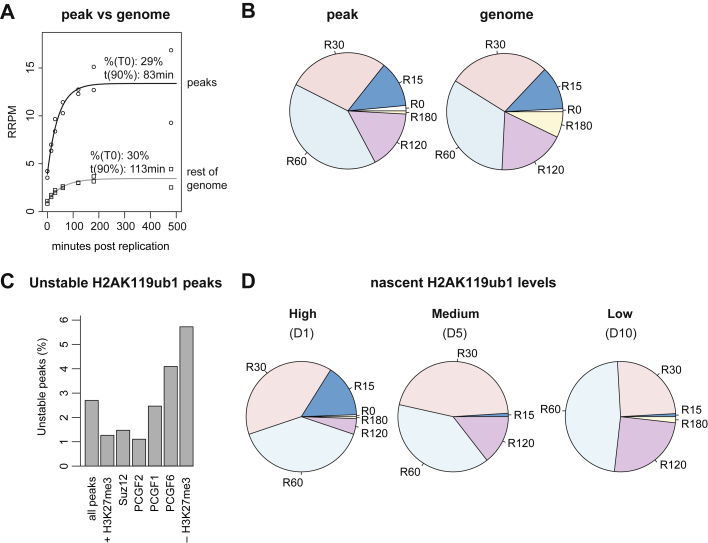
Variant PRC1 sites show fastest H2AK119ub1 restoration, related to Figure 6 (A) Restoration curve for H2AK119ub1 peaks (parsed into 1 kb windows) or 1 kb windows not overlapping H2AK119ub1 peaks. All windows considered. (B) Restoration pie chart for H2AK119ub1 peaks (parsed into 1 kb windows) or 1 kb windows not overlapping H2AK119ub1 peaks. Only non-zero and non-decreasing windows considered. (C) Relative abundance of unstable H2AK119ub1 peaks stratified by their co-occupancy profile with PRC1 and PRC2 subunits. Unstable peaks defined as peaks where signal is decreasing in subsequent time points. (D) Restoration pie chart show restoration categories for high, medium, and low-intensity nascent H2AK119ub1 peaks. Nascent signals were stratified into deciles according to intensity (D1, D2…D10) and the high (D1), medium (D5), and low (D10) deciles are shown. Data are represented as average of two replicates.

**Figure 6:**
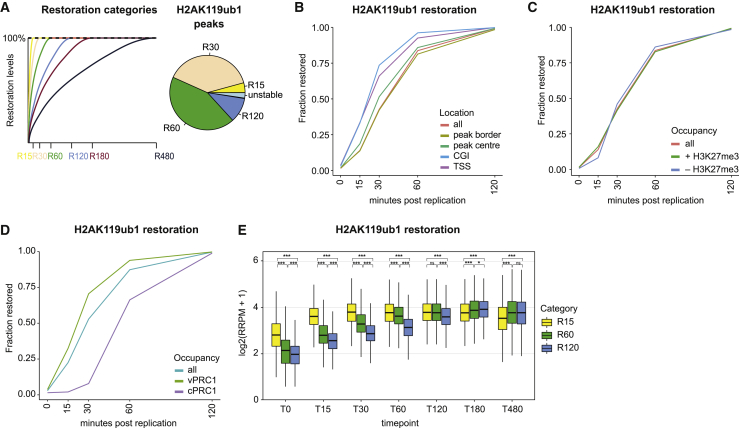
Variant PRC1 sites show fastest H2AK119ub1 restoration (A) Outline of restoration categories for H2AK119ub1 peaks. Peaks were classified as in Figure 5A (STAR Methods). (B) Restoration line plot across selected genomic features indicating the cumulative fraction restored at given time points. Parsed peaks were stratified according overlap with CpG Islands (CGI), TSSs, peak centers, or peak boundaries. (C) As in (B) but across peak regions stratified according to overlap with H3K27me3 peaks. (D) As in (B) but across peak regions overlapping either vPRC1 (PGCF1/6, not PCGF2) or cPRC1 (PGCF2, not PCGF1/6). (E) RRPM-normalized H2AK119ub1 signal over time for parsed peaks stratified according to the different restoration categories. Log_2_(RRPM + 1) scale. Statistics by unpaired Wilcoxon test. Data are represented as average of two replicates. See also Figure S6.

The interplay between H2AK119ub1 and H3K27me3 is debated, and the chronology of PRC1- and PRC2-based repression remains unresolved, especially post-replication. To address this question, we sought to identify features influencing H2AK119ub1 restoration kinetics. H2AK119ub1 is enriched both in Polycomb domains containing H3K27me3 and at PRC1 binding sites without H3K27me3.^52^ In addition, lower levels of H2AK119ub1 are distributed over large genomic regions.^33^^,^^34^^,^^53^ Comparison of restoration kinetics between high occupancy sites (called as peaks) and other genomic regions revealed that H2AK119ub1 peaks restored faster than genome-wide H2AK119ub1, also when excluding background signal across the genome (Figures S6A and S6B). Hence, H2AK119ub1 restoration is not homogeneous, but accelerated in peak regions. Next, we divided H2AK119ub1 peaks into four categories based on restoration time (Figure 6A) and unstable peaks showing decreasing levels across the time course. H2AK119ub1 levels restored with fastest kinetics at regions overlapping CpG Islands and TSSs, and peak centers were faster than peak boundaries (Figure 6B). This favors a restoration model with focused PRC1 recruitment to nucleation centers and subsequent spreading and genome-wide deposition of H2AK119ub1, similar to the proposed deposition model for H3K27me3.^16^^,^^54^

Further dissection of the features associated with H2AK119ub1 restoration revealed that H3K27me3 occupancy correlated with H2AK119ub1 stability, illustrated by a lower number of peaks losing signal across the time course in H3K27me3 regions (Figure S6C). However, the presence of H3K27me3 did not accelerate H2AK119ub1 restoration (Figure 6C). Rather, occupancy of different PRC1 subcomplexes, vPRC1 and canonical PRC1 (cPRC1), discriminated restoration kinetics. vPRC1 contains one of the four PCGF proteins 1/3/5/6 and the H2AK119ub1-reader RYBP, providing a read-write module.^20^^,^^55^ cPRC1 is characterized by PCGF proteins 2/4 and CBX proteins that read H3K27me3.^56^ Noticeably, presence of vPRC1 accelerated H2AK119ub1 restoration kinetics compared with sites occupied by cPRC1 (Figure 6D), arguing that vPRC1 recruitment with H2AK119ub1 read-write potential drives the earliest wave of H2AK119ub1 restoration. Consistent with this, restoration speed was closely linked to nascent H2AK119ub1 levels with fast-restoring peaks showing the highest H2AK119ub1 levels in nascent chromatin (Figure 6E). Reciprocally, peaks with high nascent H2AK119ub1 levels restored faster than medium- and low-intensity peaks (Figure S6D). We conclude that the rapid first wave of H2AK119ub1 restoration takes place at vPRC1-binding sites, potentially fueled by their read-write mechanism that spreads from nucleation sites to domain boundaries.

### Cross talk with H2AK119ub1 drives H3K27me3 restoration

**Figure S7:**
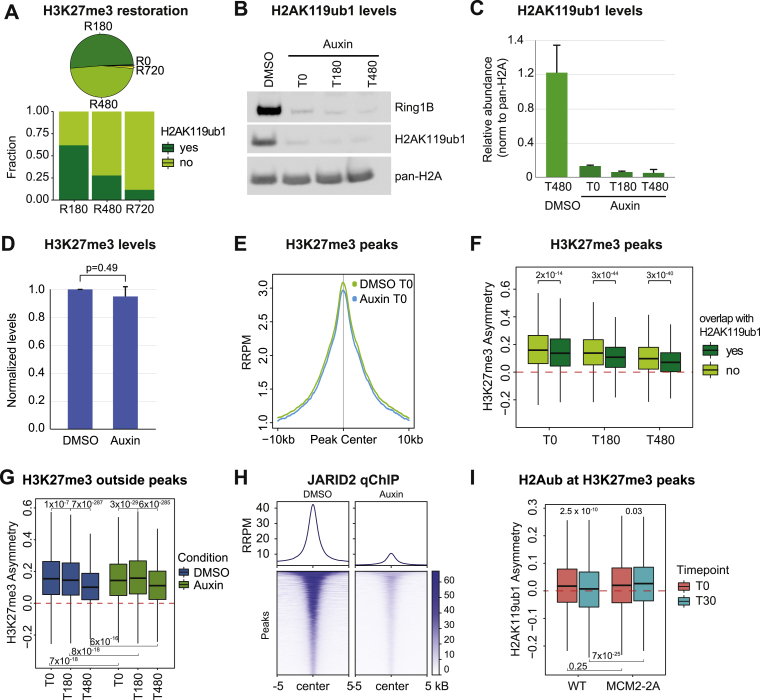
Cross talk with H2AK119ub1 drives H3K27me3 restoration, related to Figure 7 (A) (Top) ChOR-seq restoration categories for H3K27me3 in WT cells, classified identically as in Figure 5A (STAR Methods). (Bottom) Fraction of H3K27me3 peaks (parsed into 1 kB windows) overlapping H2AK119ub1ub peaks. (B) Western blot analysis of RING1B and H2AK119ub1 levels in DMSO or Auxin-treated cells (using H2A as a loading control). (C) Quantification of (B) relative to H2A. Average of two independent replicates. Quantification using ImageJ. (D) Barplot of mapped reads relative to spike-in for H3K27me3 in 2 h DMSO or Auxin-treated cells. (E) Average profile of RRPM-normalized occupancy signal for H3K27me3 across peak centers in DMSO or Auxin-treated cells. (F) Asymmetry plot showing the restoration of H3K27me3 asymmetry in DMSO-treated MCM2-2A cells over time in across H3K27me3 peaks stratified according to overlap with H2AK119ub1. (G) Asymmetry plot showing the restoration of H3K27me3 asymmetry in DMSO-treated MCM2-2A cells over time across all regions with significant signal (RPM > 0.6) but outside Polycomb domains (neither H2AK119ub1 nor H3K27me3 peaks). (H) Heatmap and average profile of total ChIP JARID2 occupancy across peak centers in genome-wide chromatin in 2.5 h DMSO or Auxin-treated cells. RRPM scale. (I) Asymmetry plot showing the restoration of H2AK119ub1 asymmetry in WT or MCM2-2A cells over time across all H2AK119ub1 peaks stratified according to overlap with H3K27me3 peaks. (F, G, and I) Statistics by unpaired Wilcoxon test. Data are represented as average of three replicates. (D) Statistics by Student t test (unpaired, two replicates).

**Figure 7:**
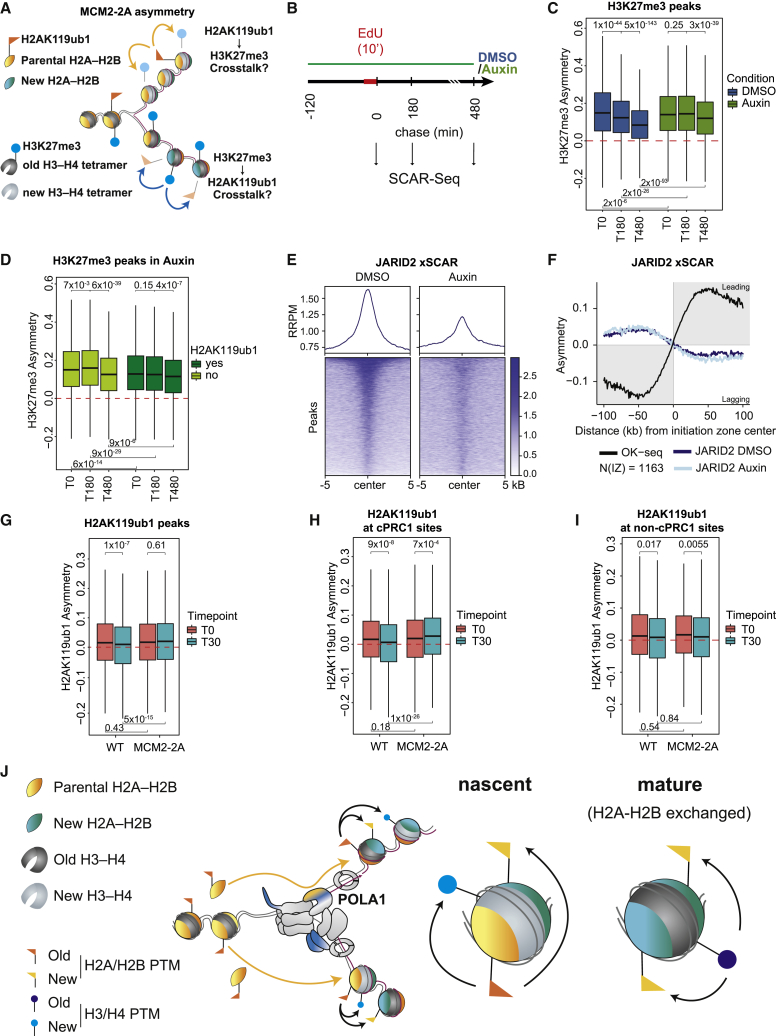
Cross talk with H2AK119ub1 drives H3K27me3 restoration (A) Illustration of the questions addressed. Does H2AK119ub1 stimulate H3K27me3 restoration on the lagging strand? Does H3K27me3 increase H2AK119ub1 deposition on the leading strand? (B) Experimental outline to assess H2AK119ub1-dependence of H3K27me3. AID-RING1B RING1A^−/−^ MCM2-2A cells were treated for 2 h with Auxin or DMSO prior to 10 min EdU labeling and indicated chase time course. (C) Asymmetry plot showing restoration of H3K27me3 asymmetry across H3K27me3 peaks in DMSO and Auxin-treated cells over time. Positive values indicate leading strand bias. Negative values indicate bias toward lagging strand. (D) Asymmetry plot showing restoration of H3K27me3 asymmetry stratified by H2AK119ub1 co-occupancy in Auxin-treated cells over time. (E) JARID2 occupancy across JARID2 peaks on nascent chromatin in untreated and Auxin-treated cells. RRPM scale. (F) Average SCAR-seq profile of JARID2 in untreated or Auxin-treated cells. (G) Asymmetry plot showing restoration of H2AK119ub1 asymmetry across H2AK119ub1 peaks in WT or MCM2-2A cells over time. (H) As in (E) but focusing on H2AK119ub1 peaks overlapping cPRC1 sites (RING1B, PCGF2). (I) As in (E) but focusing on H2AK119ub1 peaks overlapping vPRC1 sites (RING1B, no PCGF2). (C, D, and G–I) Statistics by unpaired Wilcoxon test. Data are represented as average of three replicates. (J) Model. (Left) Recycling of H2A-H2B at replication forks transmits modifications on parental H2A-H2B to nascent chromatin. Parental H2A-H2B dimers are recycled to both daughter strands independent of H3-H4 tetramers through a pathway involving POLA1 on the lagging strand. Upon reincorporation on daughter strands, parental-modified H2A-H2B can form nucleosomes with new and parental H3-H4 tetramers and new H2A-H2B dimers. Fast restoration kinetics of H2A-H2B modification together with intra- and inter-nucleosomal PTM cross talk then contribute to establishment of modifications on new H3-H4 and H2A-H2B and maintenance of chromatin states across replication. (Right) We propose that recycled parental H2A-H2B PTMs guide the restoration of newly deposited H3-H4 and H2A-H2B in nascent chromatin. Vice versa, stable H3-H4 PTMs reinforce H2A-H2B modifications in mature chromatin responding to dynamic exchange of H2A-H2B. Replisome components involved in histone recycling are colored in blue for H3-H4 and yellow for H2A-H2B. Yellow arrows depict the path of H2A-H2B at the replication fork, while black arrows indicate positive feedback. See also Figure S7.

We next addressed whether recycling and fast restoration of H2AK119ub1 feeds into restoration of more stable H3K27me3. H2AK119ub1 was enriched at fast-restoring H3K27me3 domains, consistent with positive feedback (Figure S7A). To separate cross talk with H2AK119ub1 from H3K27me3 read-write stimulation, we turned to the MCM2-2A H3-H4 recycling mutant. In this model, H3K27me3 is strongly asymmetric after DNA replication, biasing the H3K27me3 read-write mechanism to the leading strand (Figure 7A). However, H3K27me3 is gradually restored over time on the lagging strand (A.W. and A.G., unpublished data), indicating that read-write based propagation is not the only driving force of H3K27me3 restoration. To evaluate the importance of H2AK119ub1 in restoration of H3K27me3, we engineered the MCM2-2A mutation into RING1B-degron mESCs to rapidly deplete H2AK119ub1 prior to DNA replication. We then followed the impact of H2AK119ub1 on restoration of H3K27me3 by measuring asymmetry over time by SCAR-seq (Figure 7B), as H3K27me3 establishment on the lagging strand will reduce asymmetry between the two daughter strands. Treatment for 2 h with Auxin prior to EdU labeling led to almost complete depletion of RING1B and H2AK119ub1 (Figures S7B and S7C),^32^ while H3K27me3 levels remained unaltered allowing similar levels of H3K27me3 recycling (Figures S7D and S7E). In mock-treated cells, H3K27me3 asymmetry gradually declined post-replication, reflecting H3K27me3 establishment on the lagging strand. This was especially prominent in H3K27me3 peaks overlapping H2AK119ub1, consistent with positive cross talk between H2AK119ub1 and PRC2 in restoring H3K27me3 (Figure S7F). H2AK119ub1 depletion strongly delayed restoration of H3K27me3 symmetry post-replication (Figure 7C), especially within the first 3 h. Intriguingly, in this setting H3K27me3 asymmetry increased post-replication at sites without H2AK119ub1 (Figure 7D), and a similar effect was evident at sites with lower H3K27me3 levels outside Polycomb domains (Figure S7G). Increased asymmetry indicates that PRC2 activity on the leading strand, likely converting H3K27me2 to H3K27me3, is favored over modification of histones on the lagging strand. This argues that H2AK119ub1 not only promotes H3K27me3 at H2AK119ub1-decorated sites post-replication, but also limits spurious PRC2 activity that might lead to unwanted *de novo* deposition of H3K27me3. To further dissect the role of H2AK119ub1 in recruitment of PRC2, we analyzed the occupancy of the H2AK119ub1 reader JARID2, part of PRC2.2.^57^ Removal of H2AK119ub1 reduced JARID2 occupancy in nascent chromatin (Figures 7E and S7H), revealing H2AK119ub1-dependent recruitment of JARID2 post-replication. JARID2-binding symmetry to daughter strands remained unchanged (Figure 7F), consistent with H2AK119ub1 contributing to PRC2.2 recruitment on both daughter strands.

H2AK119ub1 occupancy on daughter strands was largely unaffected by the strong H3K27me3 asymmetry in MCM2-2A cells post-replication (Figure 7G), consistent with a limited role for H3K27me3 in H2AK119ub1 restoration (Figures 6C and 6D). However, H3K27me3 asymmetry had a small effect on H2AK119ub1, delaying resolution of the mild nascent H2AK119ub1 asymmetry (Figure 7G). In MCM2-2A, H2AK119ub1 asymmetry increased 30 min post-replication across sites co-occupied by cPRC1 or H3K27me3 (Figures 7H and S7I), while H2AK119ub1 became almost symmetric at these sites in WT cells. This suggests a minor role of H3K27me3 in the slow wave of H2AK119ub1 restoration or a stabilizing effect on recycled parental H2AK119ub1 (Figure S6C). Consistent with this, H2AK119ub1 sites that do not overlap cPRC1-binding sites behaved similarly in MCM2-2A and WT cells (Figure 7I).

In conclusion, tracking cross talk of H3-H4 and H2A-H2B modifications post-replication revealed that H2AK119ub1 guides timely and accurate restoration of H3K27me3, while H3K27me3 has a very minor role in H2AK119ub1 restoration.

## Discussion

### H2A-H2B are recycled with their modifications during DNA replication

The fate of parental H2A-H2B during DNA replication has proven challenging to explore due to the higher rates of H2A-H2B exchange pre- and post-replication as compared with H3-H4. Using technologies with high temporal and spatial resolution relative to replication, we interrogated H2A-H2B modifications and variants in nascent chromatin 15 kb or less behind the replication fork across the genome. This time resolution is critical, as H2AK119ub1 and H2BK120ub1 are highly dynamic modifications. To further counteract these dynamics and exclusively track modified parental H2A-H2B, we employed rapid inhibition and targeted degradation approaches to prevent *de novo* ubiquitination of H2A and H2B post-replication. Together, these approaches showed that parental H2A-H2B are recycled with their modifications locally such that positional information is maintained after replication (Figure 7J). With this property, H2A-H2B can transmit information about chromatin states to newly synthesized DNA, contributing to chromatin restoration and epigenetic cell memory.

H2A-H2B are recycled in a manner that maintains H2AK119ub1 Polycomb domain structure, H2A.Z demarcation of TSSs and H2BK120ub1 decoration of gene bodies. This is remarkably like H3-H4 recycling that maintains H3K27me3 domains, H3K4me3 promoter demarcation and H3K36me3 decoration of gene bodies.^16^^,^^23^^,^^24^ This argues that H2A-H2B recycling is acting genome-wide, during replication of both eu- and heterochromatin. Similar to H3-H4 recycling,^23^^,^^24^ parental H2A-H2B are also segregated to both daughter strands with a small leading strand bias and are redeposited with similar accuracy to H3-H4. Intriguingly, the mechanism responsible for H2A-H2B recycling is largely independent from H3-H4 recycling, with the exception that POLA1 may serve as a common histone landing pad during recycling. This is consistent with the ability of POLA1 to bind both H2A-H2B and H3-H4,^26^^,^^45^ while MCM2 and POLE3/4 are primarily H3-H4 chaperones.^24^^,^^58^^,^^59^ This model is also consistent with studies in *Drosophila* germline stem cells showing H3-H4 are inherited asymmetrically to daughter cells whereas H2A-H2B are not.^60^

Using qChOR-seq we derived restoration kinetics remarkably similar to that obtained by mass spectrometry for H3K27me3.^18^^,^^49^ We estimated histone modifications/variants in nascent chromatin to be about 25%–35% of steady-state levels, while mass spectrometry measured recycling of old H3K27me3, H3K9me3, and H4K20me2 to be slightly below 50% and for H2A.Z around 30%.^18^ The amplification and spike-in normalization steps in qChOR-seq represents a potential limitation compared with direct measurements by mass spectrometry but allows measurements of histone recycling that maintain positional information. Importantly, the recycling efficiency of H2A-H2B modifications and H3K27me3 are in close range, while H2A.Z shows slightly lower recycling efficiency in agreement with mass spectrometry measurements. Collectively this argues that H2A-H2B are recycled accurately with comparable efficiency to H3-H4, while H2A.Z recycling might be slightly less efficient.

### H2A-H2B short-term memory promotes post-replication chromatin restoration

Chromatin restoration in the wake of replication involves a multitude of different processes acting at different timescales. While newly deposited histones are deacetylated within 10–15 min,^7^ restoration of pre-replication H3K27, H3K9, and H4K20 tri-methylation levels can take up to a full cell cycle.^18^^,^^49^ In the face of this dilution, maintenance of silencing post-replication represents a paradox in gene regulation. Based on the fast restoration kinetics of H2AK119ub1, we propose that H2AK119ub1 recycling and fast restoration re-establish repression rapidly post-replication. The H2A variant macroH2A may serve a similar function in constitutive heterochromatin.^61^^,^^62^

The fastest wave of H2AK119ub1 restoration post-replication occurs at vPRC1-bound sites, where —in addition to sequence-dependent recruitment—H2AK119ub1-based read-write mechanisms involving RYBP may act to restore H2AK119ub1^20^^,^^55^ (Figure 7J). In addition, H3K27me3 restoration kinetics and accuracy is facilitated by H2AK119ub1 shortly after replication. This finding explains why loss of H3K27me3 upon RING1A/B depletion correlates with cell division.^32^ It also agrees with PRC2.2, containing JARID2, being active mainly in G2, whereas PRC2.1 acts more in G1.^63^ Similarly, dynamic histone modifications can pave the way for stable repression during establishment of X inactivation.^64^ In epigenome maintenance, we propose that histone PTM cross talk is especially important post-replication to restore chromatin states (Figure 7J). The recycling of H2AK119ub1 serves an important role in providing short-term memory post-replication in terms of positive feedback required to prioritize appropriate loci. In this way, short-term memory of dynamic modifications can feed into the establishment of slower, long-term memory provided by H3-H4 modifications such as H3K27me3.

Early pioneering work found that new H3-H4 tetramers mainly form nucleosomes with old H2A-H2B dimers,^9^ consistent with our finding that H2A-H2B recycling is independent of H3-H4 recycling. Therefore, an added value of H2A-H2B and H3-H4 crosstalk post-replication is that, in contrast to H3-H4 read-write models, it can operate within the same nucleosome (Figure 7J). Recycling of modified H2A-H2B can embed new H3-H4 tetramers in an environment that mirrors the pre-replication state and facilitates restoration. The dynamics of H2A-H2B argues that molecules recycled during replication might not themselves be transmitted through mitosis. Their epigenetic function would be to transmit chromatin states across replication, feeding into restoration of H3-H4 modifications and thereby mitotic transmission.

### H2A-H2B memory of active states

H2A.Z and H2BK120ub1 have been linked to actively transcribed chromatin; recycling and restoration of such features may therefore maintain a chromatin landscape permissive for transcription. We show that restoration of active chromatin modifications is heavily linked to transcription re-start. However, restoration of H2BK120ub1 in bodies of long genes shows features suggesting a restoration mechanism different from RNAPII progression across the gene. Nascent chromatin harbors accessible regions in gene bodies with a strong skew toward the 3′ end.^27^ The origin of this accessibility is unclear, but the faster restoration of H2BK120ub1 in these regions renders support to the idea that cryptic transcription is involved.^27^ In terms of restoration of active chromatin states, it will be important to address how transcription re-starts after replication and the role of short-term memory by recycled H2A.Z and H2BK120ub1.

### Limitations of the study

Although we profiled multiple replisome components, only POLA1 revealed a role in H2A-H2B recycling to the lagging strand. Thus, the exact molecular components that handle H2A-H2B during DNA replication remain to be identified. Our work implies that chaperones involved in H2A-H2B recycling should accommodate ubiquitylated H2A and H2B as well as H2A.Z. For the future, H2A-H2B recycling mutants will be necessary to dissect the individual roles of H2A-H2B and H3-H4 recycling in homeostasis and differentiation. Furthermore, our study highlights the role of short-term memory by H2AK119ub1 in mESCs, a dynamic system with high activity of vPRC1 subcomplexes. This changes during differentiation, where the stoichiometry and composition of Polycomb complexes change significantly and additional regulatory elements such as phase separation take effect.^65^ Studying the function of histone H2A-H2B recycling in differentiated systems thus represents an exciting avenue to investigate epigenetic inheritance of histone modifications.

## STAR★Methods

### Key resources table

**Table:** 

REAGENT or RESOURCE	SOURCE	IDENTIFIER
**Antibodies**		

H2AK119ub (rabbit)	Cell Signaling Technology	Cat no. 8240; RRID: AB_10891618
Pan-H2A (rabbit)	Abcam	Cat no. ab177308
H2A.Z (rabbit)	Abcam	Cat no. ab188314
H2BK120ub (rabbit)	Cell Signaling Technology	Cat no. 5546; RRID: AB_10693452
H3K27me3 (rabbit)	Cell Signaling Technology	Cat no. 9733; RRID: AB_2616029
Pan-H3 (rabbit)	Abcam	Cat no. ab1791; RRID: AB_302613
H2A.V (mouse)	Active Motif	Cat no. 61751; RRID: AB_2793757
H4K20me0 (rabbit)	Abcam	Cat no. ab227804
RING1B (rabbit)	Cell Signaling Technology	Cat no. 5694; RRID: AB_10705604
BAP1 (rabbit)	Cell Signaling Technology	Cat no. 13271; RRID: AB_2798168
PCNA (mouse)	Abcam	Cat no. ab29, RRID: AB_303394
Pan-H2B (mouse)	Abcam	Cat no. ab52484, RRID: AB_1139809
JARID2 (rabbit)	Cell Signaling Technology	Cat no. 13594, RRID: AB_2798269
CW680 anti-mouse	LI-COR	RRID: AB_10953628
CW800 anti-rabbit	LI-COR	RRID: AB_621843
HRP-anti-rabbit	Jackson	Cat no. 711-035-152
HRP-anti-mouse	Jackson	Cat no. 115-035-068

**Chemicals, peptides, and recombinant proteins**		

Gelatin from bovine skin	Sigma	Cat no. G9391
DMEM	Gibco	Cat no. 31966
Fetal Calf Serum	GE Hyclone	Cat no. SV30160.03
Penicillin/ Streptomycin	GIBCO	Cat no. 151400122
Non-essential amino acids	GIBCO	Cat no. 11140050
2-Mercaptoethanol	GIBCO	Cat no. 21985023
Leukemia Inhibitory Factor	Custom-made	N/A
Shields and Sang M3 Insect Medium	Sigma	Cat no. S-8398
KHCO3	Sigma	Cat no. 60339
Yeast Extract	Sigma	Cat no. Y-1000
Bactopeptone	BD	Cat no. 211705
Lipofectamine 3000	Thermo Fisher Scientific	Cat no. L3000015
5-ethynyl-20-deoxyuridine	Invitrogen	Cat no. A10044
Triptolide (TPL)	Sigma	Cat no. T3652
DMSO	Sigma	Cat no. D2650
Indole-acetic acid (Auxin) sodium salt	Sigma	Cat no. I5148
dTAG-13 (dTAG)	Tocris	Cat no. 6605/5
Trypsin	GIBCO	Cat no. 25200056
Formaldehyde	Thermo Fisher	Cat no. 28908
Protein A agarose beads (ChOR-Seq)	Thermo Fisher	Cat no. 20333
Pierce™ ChIP-Grade Protein A/G Plus Agarose (ChOR-Seq)	Thermo Fisher	Cat no. 26161
Dynabeads™ M-280 Sheep Anti-Rabbit IgG (SCAR-Seq)	Thermo Fisher	Cat no. 11204D
Dynabeads Protein G (xSCAR-Seq)	Thermo Fisher	Cat no. 10004D
Pierce™ High Capacity Streptavidin Agarose (aniPOND)	Thermo Fisher	Cat no. 20357
10x Buffer BXT	IBA-LIFESCIENCES	Cat no. 2-1042-025
Picolyl-azide-PEG4-biotin (ChOR-Seq)	Jena Bioscience	Cat no. CLK-1167-100
Biotin-TEG-Azide ((x)SCAR-Seq)	Berry & Associates	Cat no. BT1085
THPTA	Sigma	Cat no. 762342
Agencourt AMPure XP beads	Beckman Coulter	Cat no. A63881
Dynabeads MyOne Streptavidin T1 beads	Invitrogen	Cat no. 65602
RNase A	Sigma	Cat no. R4875
Proteinase K	Sigma	Cat no. P6556

**Critical commercial assays**		

truChIP Chromatin Shearing Kit	Covaris	Cat no. 520127
MinElute PCR Purification Kit	QIAGEN	Cat no. 28004
Click-iT EdU Kit	Thermo Fisher	Cat no. C10337
MinElute Reaction Cleanup Kit	QIAGEN	Cat no. 28204
KAPA Hyperprep Kit	Kappa Biosystems, Roche	Cat no. KK8504
DNA Kit for Bioanalyzer	Agilent	Cat no. 5067-4626

**Deposited data**		

Raw and analyzed data	This paper	GEO: GSE204988
Western Blot Images	This paper	Mendeley Data: https://doi.org/10.17632/7pxmj6dnp9.1
mm10 Blacklist	ENCODE Project Consortium, 2012	http://mitra.stanford.edu/kundaje/ akundaje/release/blacklists/mm10mouse/mm10.blacklist.bed.gz
Okazaki-Seq Peak set	Petryk et al.^23^	GEO: GSE117274
H3K4me3 Raw data (datasets: ENCFF997CAQ, ENCFF425ZMW)	Sethi et al.^51^	https://doi.org/10.17989/ENCSR000CGO
RNAPII-Ser5p Raw data	Stewart-Morgan et al.^27^	GEO: GSE128643
Enhancer Peak set	The FANTOM consortium et al.^66^	http://doi.org/10.1186/s13059-014-0560-6
CpG Islands Peak set	Illingworth et al.^67^	GEO: GSE21442
RING1B, SUZ12 Peak sets	Blackledge et al.^68^	GEO: GSE132752
PCGF1/2/6 Raw data	Blackledge et al.^68^	GEO: GSE132752

**Experimental models: Cell lines**		

Mouse: E14 ES cells (WT)	Laboratories of K. Helin and J. Brickman	RRID: CVCL_C320
*Drosophila* S2-DRSC cells	Drosophila Genomics Resource Center	Stock No. 181; RRID: CVCL_Z992
HCT116 cells	Laboratory of A. Lund	RRID: CVCL_0291
Mouse ESC: AID-RING1B;RING1A-/-	Rhodes et al.^31^	N/A
Mouse ESC: AID-RING1b;RING1A-/- BAP1-dTAG	This study	N/A
Mouse ESC: AID-RING1B;RING1A-/- MCM2-2A	This study	N/A
Mouse ESC: POLA1-3A	This study	N/A
Mouse ESC: MCM2-2A	This study	N/A
Mouse ESC: POLE4KO	This study	N/A

**Oligonucleotides**		

Oligonucleotides	This study	See Table S1
TALEN target sequences	This study	See Table S1
PentAdapters for Illumina NGS	Pentabase	Cat no. 316
xGen UDI-UMI Adapters, 1-96, set	Integrated DNA Technologies	Cat no. 10005903

**Software and algorithms**		

Trim Galore! v0.0.6	Babraham Bioinformatics	https://www.bioinformatics.babraham.ac.uk/ projects/trim_galore/
Bowtie2 v2.4.2	Langmead and Salzberg^69^	https://github.com/BenLangmead/bowtie2
SAMtools v1.12	Li et al.^70^	http://www.htslib.org/
Picard MarkDuplicates v2.26.10	“Picard Toolkit.” 2019. Broad Institute	https://github.com/broadinstitute/picard/releases/tag/2.27.3
MACS2 v2.2.6	Zhang et al.^71^	https://github.com/taoliu/MACS/tree/ master/MACS2
deepTools v.3.5.1	Ramírez et al.^72^	https://deeptools.readthedocs.io/en/ develop/
BEDtools v2.30.0	Quinlan^73^	https://bedtools.readthedocs.io/en/latest/
Seqmonk v1.47.1	Babraham Bioinformatics	https://www.bioinformatics.babraham.ac.uk/ projects/seqmonk/
SeqPlots v.12.1	Stempor and Ahringer^74^	https://bioconductor.org/packages/release/bioc/html/ seqplots.html
R v4.1.2	R Project	https://www.r-project.org/
Bioconductor	Huber et al.^75^	https://www.bioconductor.org/
UCSC LiftOver	UCSC Genome Browser	https://genome.ucsc.edu/cgi-bin/hgLiftOver
wigtoBigwig	UCSC Genome Browser	http://hgdownload.cse.ucsc.edu/admin/exe/
ImageJ v1.53k	Schneider et al.^76^	https://imagej.net/software/imagej/
SCAR-Seq Analysis Pipeline	Petryk et al.^23^	https://github.com/anderssonlab/Wenger_et_al_2022

### Resource availability

#### Lead contact

Further information and requests for resources and reagents should be directed to and will be fulfilled by the lead contact Anja Groth (anja.groth@cpr.ku.dk).

#### Materials availability

Oligonucleotides, targeting constructs for genome editing and newly generated cell lines are available upon request and should be directed to lead contact Anja Groth.

### Experimental model and subject details

#### Cell Culture

WT and RING1A-/- AID-RING1B Rosa26::OsTir1 murine embryonic stem cells (mESCs) were a gift from the Helin and Klose labs,^31^ respectively and are both derived from a male E14 background. mESCs were grown on feeder-free plates coated with 0.1% gelatine in DMEM media supplemented with 15% FBS (Invitrogen), 1x penicillin/streptomycin, 1x non-essential amino acids, 1x beta-mercaptoethanol, and custom-made LIF at 37°C and 5% CO_2_. Cells were passaged using Trypsin-EDTA or TriplE and regularly tested for the absence of mycoplasma.

*Drosophila* S2-DRSC cells were obtained from the Drosophila Genomics Research Centre. S2 cells were grown in suspension in spinners in M3+BPYE media: Shields and Sang M3 Insect Medium (Sigma, S-8398), KHCO3 (Sigma, 12602), yeast extract (Sigma, Y-1000), bactopeptone (BD, 211705), 10% heat-inactivated FBS (GE Hyclone, SV30160.03) and 1X penicillin/streptomycin (GIBCO, 151400122). Cells were incubated at 25°C with 5% CO_2_.

HCT116 cells were used from Bao et al.^77^ and grown in DMEM media supplemented with Glutamax (35050061, Gibco), 10% FBS and 1x penicillin/streptomycin at 37°C and 5%CO_2_.

#### Protein Degradation and Triptolide treatment

mESCs were plated on 15 cm dishes two days prior to treatment. Degradation was induced by adding DMSO-dissolved Auxin sodium salt (IAA, Sigma) at a final concentration of 500 μM, dTAG-13 (Tocris) to 1 μM or a volume-equivalent amount of DMSO as control. For triptolide treatment, DMSO-dissolved triptolide (Sigma) was added to a final concentration of 10 μM or a volume-equivalent amount of DMSO as control for the indicated time.

#### DNA Labelling

Samples were labelled with medium containing 5-ethynyl-20-deoxyuridine (EdU; Invitrogen, A10044) at a final concentration of 10 μM for 10 minutes (30 minutes for JARID2 experiments). After labelling, nascent samples were immediately processed. All chased samples were washed twice in PBS and further incubated in fresh, unlabelled medium for the appropriate time interval before collection. For use in ChOR-Seq, *Drosophila* S2 cells were labelled with 10 μM EdU for 39 hours before further processing.

### Method details

#### Genome editing

Used oligonucleotides and TALEN target sequences are listed in Table S1.

Pola1-3A cells were generated in WT mESCs by mutating amino acids D83, W84 and D88 to alanine and an additional silent mutation of V86 and D87 (to create a SalI restriction site for screening purposes) using a TALEN protocol described in Flemr and Bühler.^78^ Briefly, TALENs were assembled using Golden Gate Cloning^79^ (Addgene # 1000000024) and acceptor vectors SV40-ELD and SV40-KKR. Cells were transfected with TALEN-EED, TALEN-KRR, the recombination reporter (pRR-Puro or pRR-eGFP) and single-stranded oligonucleotide donor (Integrated DNA technologies) using Lipofectamine 3000 reagent (Invitrogen). A transfection with one TALEN only served as a negative control. For the experiment with the pRR-Puro reporter, cells were seeded sparsely on a 10 cm 24 hours post transfection and selected with Puromycin (2 μg/mL) for 36h. After one week in culture, individual clones were picked manually with a pipette and each clone was distributed to a 96-well plate. 2 days later, the 96-well plate was split onto 3 x 96-well plates (one plate each for expansion, genotyping and backup). For transfection with the pRR-eGFP reporter, GFP-positive cells were sorted into 96-well plates (BD FACSAria III cell sorter) 24 hours after transfection and cultured for one week before expansion and genotyping.

For genotyping, cells were washed in PBS and placed at -80°C for >30 minutes. Subsequently, cells were scraped off with 50 μL squishing buffer (10 mM Tris pH 8, 1 mM EDTA, 25 m MNaCl, 200 μg/mL Proteinase K), transferred to PCR tubes and incubated for 1 hour at 65°C followed by 10 minutes at 95°C. The resulting gDNA was genotyped using One Taq Hot Start 2x Master Mix (NEB) and indicated primers. Half of the product was digested with restriction enzyme SalI (NEB) to identify positive clones and subsequently Sanger sequenced.

MCM2-2A cells were generated in RING1A-/- AID-RING1B cells following the detailed protocol from Petryk et al.^23^

BAP1-dTAG cells were generated in RING1A-/- AID-RING1B cells by CRISPR-Cas9 using the SpCas9(BB)-2A-Puro (PX459) V2.0 plasmid (Addgene #62988) as described in Blackledge et al.^68^ with sgRNA#1, targeting the *Bap1* gene at the end of the ORF and a BAP1-linker-dTAG homology donor plasmid (a gift from the Klose lab). Similarly, POLE4KO cells were generated using sgRNA#2 and sgRNA#3, targeting the *Pole4* gene upstream of exon 1 and downstream of exon 2, respectively, thereby deleting most of the ORF. Cells were transfected using Lipofectamine 3000 using 0.5μg of the sgRNA-plasmid and 2 μg of the donor plasmid. Cells were sparsely seeded on a 10 cm dish 24 hours post transfection and selected with Puromycin (2 μg/mL) for 48 h. Thereafter, cells were expanded, genotyped and analysed by Sanger sequencing as described above.

#### Western Blotting

Cells were washed once with PBS and LSB buffer was added directly to the well. Lysed cells were scraped off into an Eppendorf tube, 25U Benzonase (Sigma) was added and lysate incubated for 1 hour at 37°C before being boiled for 10 minutes at 95°C. SDS-PAGE and Western Blotting was subsequently performed as described in Petryk et al.^23^

Primary antibodies were used in TBST & 5% milk at the following concentrations: rabbit-anti-H2AK119ub1 (1:5’000), rabbit-anti-H2A (1:5’000), rabbit-anti-Ring1B (1:1’000), rabbit-anti-BAP1 (1:1’000), rabbit anti-H2A.Z (1:5’000), mouse anti-H2B (1:5’000), mouse anti-PCNA (1:1’000), rabbit-anti-H2BK120ub1 (1:2’000) and incubated with the membrane on a roller overnight at 4°C. The next day, membranes were washed 3x with PBST, incubated with an HRP-conjugated secondary antibody (1:15’000) for 1 hour at room temperature (RT), followed by 3x washes with PBST before signal detection. Signal was detected with Pierce ECL or ECL Pico Plus Western Blotting Substrate (ThermoScientific) with a ImageQuant LAS 4000 Camera (GE Healthcare) and signal intensity quantified using ImageJ (Version 1.53k^76^). For aniPOND-WB, CW800 anti-rabbit or CW680 anti-mouse secondary antibodies were used and detected with an Odyssey LI-COR machine.

#### ChOR-Seq & ChIP-Seq

A step-by-step protocol is available in Petryk et al.^43^ For ChIP-Seq and ChOR-Seq, 4.5 x 10^6^ mESCs or 1 x 10^7^ HCT116 cells were seeded in a 15 cm plate (2-3 plates per timepoint). Two days later, following appropriate treatment (EdU label or chase), cells were washed twice with PBS and once with cold 1x PBS. Subsequently, 7mL Fixation buffer A (truChIP Chromatin Shearing Kit, Covaris, 520127) was added to each plate and 11.1% fresh formaldehyde added to a final concentration of 1% with constant movement at RT for 5 minutes (for histones PTMs) or 10 minutes (for RNAPII and RING1B). Then, glycine was added to a final concentration of 0.1 M and the reaction was quenched for 5 minutes with constant movement at RT. Fixed cells were washed twice in ice-cold PBS and then collected in ice-cold PBS using a cell lifter before centrifuging for 5 minutes at 500g at 4°C. Cells were transferred to Eppendorf tubes and centrifuged 5 minutes at 500g at 4°C. Cell pellets were snap-frozen in liquid nitrogen and stored at -80°C until lysis.

Nuclei isolation was performed on fixed cells using the truChIP Chromatin Shearing Kit (Covaris, 520127) following manufacturer’s instructions. 2 x 10^7^ cells were sonicated in 1 mL tubes using a Covaris S220 with the following settings: duty cycle 10% intensity, 200 cycles/ burst, 20 minutes processing time, 7°C bath temperature, water level full. Sonicated chromatin was centrifuged at 14,000 rpm at 4°C for 10 minutes and the supernatant was used for subsequent steps. In parallel, *Drosophila* S2 cells were fixed, lysed, and sonicated as described above. After sonication, input chromatin was mixed with EdU-labelled *Drosophila* S2 chromatin (0.05% of total chromatin). For each mark and timepoint ChIP, 2 x 25 μg total of mixed mESCs or HCT116 cells and *Drosophila* S2 sonicated chromatin were diluted up to 500 μL with dialysis buffer (4% glycerol, 10 mM Tris-HCl, 1 mMEDTA, 0.5 mM EGTA; pH 8) and 400 μL of incubation buffer (2.5% Triton X-100, 0.25% sodium deoxycholate, 0.25% SDS, 0.35 M NaCl, 10 mM Tris-HCl; pH 8) supplemented with leupeptin, aprotinin, pepstatin, and PMSF. Only 1x 25 μg chromatin was used for pan-H2A and pan-H3 ChIPs. Chromatin was pre-cleared with Protein A Agarose beads (Thermo) for 1 hour at 4°C. After pre-clearing, 10 μL were set aside as Input and the remaining chromatin was incubated overnight at 4°C with rotation with the indicated amount of antibody (each 25 μg chromatin with following amounts of antibody: 10 μL H2AK119ub1, 10 μL H2BK120ub1, 20 μL H3K27me3, 2 μg pan-H2A, 2 μg pan-H3). Protein A Agarose beads were incubated in 1 mg/ml BSA in RIPA buffer overnight at 4°C with rotation. The next day, the chromatin-antibody mix was incubated for 3 hours with the pre-blocked Protein A Agarose beads.

For H2A.Z, chromatin was incubated with 3 μg H2A.Z antibody and 0.2 μg H2A.V antibody to ensure *Drosophila* chromatin capture for Spike-In reference as H2A.Z is not conserved between mammals and flies. Protein A/G Plus Agarose beads (Thermo) were used instead for preclearing and antibody capture.

Subsequently, ChIPs were washed three times in ice-cold RIPA buffer (140 mM NaCl, 10 mM Tris-HCl, 1 mM EDTA, 1% Triton X-100, 0.1% SDS, 0.1% sodium deoxycholate, 1 mM PMSF; pH 8), three times in RIPA buffer with 0.5 M NaCl, once in LiCl buffer (250 mM LiCl, 10 mM Tris-HCl, 1 mM EDTA, 0.5% IGEPAL CA-630, 0.5% sodium deoxycholate; pH 8) and twice in TE (10 mM Tris-HCl, 1 mM EDTA; pH 8). The set-aside inputs and the washed beads were incubated with 50 μg RNase A (Sigma) for 30 minutes at 37°C, thereafter SDS and NaCl were added to a final concentration of 0.5% and 100mM, respectively and samples were incubated with proteinase K (10 μg) for 10 hours at 37°C followed by 6 hours incubation at 65°C for de-crosslinking. DNA was purified using the MinElute PCR purification kit (QIAGEN, 28004).

For RING1B qChOR-seq, 2 x 50 μg of chromatin (mixed with 0.05% of *Drosophila* S2 chromatin) was used per timepoint and incubated each with 30 μL RING1B and 2 μL H2A.V antibodies. Protein A/G Plus Agarose beads (Thermo) were used instead for preclearing and antibody capture. Subsequently, the beads were washed four times with ice-cold RIPA buffer, once with ice-cold RIPA buffer with 0.5 M NaCl, once in LiCl buffer and once in TE. Decrosslinking and DNA purification was performed as indicated above.

For total ChIPs, 5% of the immunoprecipitated DNA (or maximum 5-10 ng) was subjected to end repair, A-tailing and amplification using the KAPA Hyperprep kit protocol (Roche, KK8504) and Illumina-compatible indexed adapters (Pentabase, SKU 310). Before amplification, DNA was size selected with Agencourt AMPure XP beads (Beckman Coulter, A63881) to obtain fragments between 200-700 bp. For amplification, 5-7 PCR cycles were used followed by clean-up with Agencourt AMPure XP beads.

For qChOR-Seq experiments, immunoprecipitated DNA from two ChIP reactions were pooled for each time point, except pan-H2A and pan-H3 where only one ChIP reaction was performed. Then, all immunoprecipitated DNA (without the aliquot set-aside for total ChIP, see above) or 20 ng de-crosslinked input material was subjected to end repair, A-tailing, and adaptor ligation using the KAPA Hyperprep kit following manufacturer’s instructions and cleaned-up with Agencourt AMPure XP beads. For qChOR-Seq experiments, indexed DNA from all time points in the same time course were then pooled prior to click biotinylation. Then, click reaction was performed on indexed and mixed DNA for 30 minutes at RT under the following conditions: 1X Click-IT buffer (Click-iT EdU Alexa Fluor 488 Imaging Kit, Thermo Fisher, C10337), 0.5 mM picolyl-azide-PEG4-biotin (Jena Bioscience, CLK-1167-100), 0.1 mM CuSO4 (from Click-iT kit), 0.5 mM THPTA (Sigma, 762342), and 10 mM sodium ascorbate (from Click-iT kit). DNA fragments were purified using Agencourt AMPure XP beads and resuspended in elution buffer (10 mM Tris-HCl pH 8.5). Next, to capture biotinylated products, MyOne Streptavidin T1 beads (Invitrogen, 65602) were washed three times with 1X B&W buffer (5 mM Tris-HCl pH 7.5, 0.5 mM EDTA, 1 M NaCl, 0.05% (V/V) Tween-20) and resuspended in 2X B&W buffer at a volume equal to the volume of biotinylated DNA. Streptavidin beads were then mixed with biotinylated DNA and rotated for 30 minutes at RT. Beads containing biotinylated DNA were washed four times with 1X B&W buffer, twice with 1X TE with 0.05% (V/V) Tween 20, and once with 10 mM Tris-HCl pH 7.5. Finally, beads were resuspended in elution buffer (10 mM Tris-HCl pH 8.5).

PCR amplification of ChOR-Seq samples was performed following the KAPA Hyperprep kit protocol using the streptavidin bead suspension as a template (9-12 cycles of PCR). Following PCR, streptavidin beads were purified using a magnetic rack, and the supernatant was cleaned-up twice with Agencourt AMPure XP beads, once with size selection to obtain fragments between 200-700 bp and once at a ratio of 1.0x. Fragment distribution of libraries was checked on a Bioanalyzer using the high sensitivity DNA kit (Agilent) or a Fragment Analyzer system (Agilent) and libraries were pooled and sequenced on a NextSeq500 or NextSeq2000 instrument (Illumina).

All samples and sequencing parameters are listed in Table S2.

#### SCAR-Seq

A step-by-step protocol is available in Petryk et al.^43^ Cells were seeded in 15 cm dishes (4.5 x 10^6^ cells per dish) 2 days prior to EdU labelling and nuclei isolation. After a 10 minutes 10 μM EdU pulse, nascent samples were harvested immediately. Chase samples were washed two times with PBS and incubated in media for 30 minutes (T30), 3 hours (T180) or 8 hours (T480) before harvesting. For sample collection, media was aspirated, plates washed 2x with RT PBS and ice-cold PBS was added to the dishes. Cells were scraped in a cold room and collected by centrifugation, followed by nuclei isolation. Nuclei were aliquoted, snap-frozen and stored at -80°C until further use. For MNase digest, nuclei were counted manually using Kova Glasstic Slides and 2 U MNase (Worthington) were added per 1 x 10^6^ nuclei. Digests were performed at 30°C for 20 minutes.

For native ChIP, 30-50 μg of chromatin was used per sample and incubated with antibodies in a total volume of 600 μL overnight at 4°C (40 μg chromatin with 10 μL H2AK119ub1, 30 μg chromatin with 10 μL H3K27me3, 25 μg chromatin with 4 μL H4K20me0 antibodies). Magnetic beads (anti-rabbit IgG Dynabeads, invitrogen) were added the next morning and samples were incubated for 2 hours at 4°C. Subsequently, ChIPs were washed three times with ice-cold RIPA buffer and three times with ice-cold RIPA buffer with 0.5 M NaCl. DNA was eluted and purified using the MinElute Reaction Cleanup kit (Qiagen). Mononucleosomal-sized fragments were isolated by double sided size selection with AMPure XP beads. 5 % of the purified DNA sample was set aside to prepare libraries for quantitative total ChIP-seq library preparation. The remaining EdU-labelled DNA fragments were biotinylated using click chemistry as reported above but using Biotin-TEG-Azide (Berry & Associates) instead of Picolyl-azide-PEG4-Biotin.

Libraries were prepared using the KAPA Hyper Prep Kit (Roche). Biotinylated fragments were captured using Dynabeads MyOne Streptavidin (invitrogen) and EdU-labeled strands were isolated by performing NaOH washes. SCAR-seq libraries were amplified using 9 to 11 PCR cycles, while 5 cycles were used for total ChIP samples. Libraries with mononucleosomal-sized inserts were isolated by double-sided size selection with AMPure XP beads, followed by a second clean-up with 1.0x AMPure XP beads. Fragment distribution of libraries was checked on a Bioanalyzer using the high sensitivity DNA kit (Agilent) or a Fragment Analyzer system (Agilent). Stranded input samples were prepared for all cell lines and time points in parallel with SCAR-Seq samples. Libraries were pooled and sequenced on a NextSeq500 instrument (Illumina). All samples and sequencing parameters are listed in Table S2.

#### xSCAR-Seq

Crosslinked SCAR-seq (xSCAR-seq) was conducted identically to ChOR-seq with the following changes: For H2A.Z, 120 μg chromatin was used per sample and incubated with 16 μg antibody. For H4K20me0, 30 μg chromatin was used and incubated with 4 μg antibody. As for ChOR-seq input chromatin was mixed with EdU-labelled *Drosophila* S2 chromatin (0.05% of total chromatin). All steps up to streptavidin pull down of biotinylated DNA were performed following the ChOR-seq protocol, except that Biotin-TEG-Azide was used in the click reaction instead of Picolyl-azide-PEG4-Biotin. Biotin-labelled DNA fragments were then purified and amplified following the procedure for SCAR-seq, including the three crucial 100 mM NaOH washes to isolate nascent EdU-labeled strands.

For JARID2 and xSCAR-seq, mES cells were labelled for 30 minutes with 10 μM EdU and harvested identically to the ChOR-seq procedure above with the following changes. 150 μg chromatin (mixed with 0.05% of *Drosophila* S2 chromatin) was subjected to immunoprecipitation using 50 μL JARID2 antibody and 0.8 μg H2A.V antibody for Spike-In reference. After rotation overnight at 4°C, the mixture was captured with Protein G Dynabeads rotating for 2 hours at 4°C. Beads were washed three times with ice-cold RIPA buffer and once with ice-cold RIPA buffer with 0.5 M NaCl before eluting with 1% SDS in TE. Decrosslinking and DNA purification was performed as indicated above for ChOR-seq. 5% of the eluted material was used to construct total ChIP-seq libraries, while the rest was subjected to library preparation, click biotinylation, and Streptavidin pulldown according to the SCAR-seq protocol outlined above. Stranded and total Input libraries were constructed for all samples in parallel. Samples were pooled and sequenced on a NextSeq500 (Illumina). All samples and sequencing parameters are listed in Table S2.

#### native iPOND (aniPOND)

The native iPOND (aniPOND) procedure was broadly based on the protocol from Wiest and Tomkinson.^80^ In brief, 3 x 15 cm plates per condition corresponding to approx. 1.5 x 10^8^ mES cells were harvested after 10 minutes 10 μM EdU labelling (the noEdU control omitted this step), nuclei extracted and snap-frozen in buffer A according to the SCAR-seq procedure. Cells were thawed on ice, resuspended in 10ml ice-cold PBS and subjected to click biotinylation in a final volume of 5 mL (1x PBS, 10 μM picolyl-azide-PEG4-biotin, 10 mM Sodium ascorbate, 2 mM Copper sulfate, 100 μM THPTA) for 60 minutes at 4°C. After the click reaction, nuclei were spun down at 1300g for 10 minutes at 4°C and washed once with PBS. Then, nuclei were resuspended in Buffer B1 (25 mM NaCl, 2 mM EDTA, 50 mM Tris pH 8, 1% IGEPAL, protease inhibitors), rotated for 30 minutes at 4°C and spun down at 1300g for 10 minutes at 4°C. Nuclei were resuspended again in Buffer B1, rotated for 30 minutes and subsequently sonicated in a Bioruptor (20sec ON, 40sec OFF, 15x, Power: High). Post sonication, samples were centrifuged at 16,100g for 10 minutes at 4°C and supernatant transferred to a new tube. 10 μL of the supernatant were used to determine the DNA concentration and used to normalize the samples to equal DNA amounts. 30 μL of the equalized supernatant were set aside as Input, lysed in LSB buffer (1x final) and stored at -20°C. 100 μL Streptavidin beads (Thermo Fisher) per sample were washed 3x with buffer B1 and taken up in 100 μL B1 buffer and added to the equalized supernatant. The beads-protein mixture was rotated at 4°C overnight and then washed twice with Buffer B2 (150 mM NaCl, 2 mM EDTA, 50 mM Tris pH 8.0, 0.5% IGEPAL), once with 1 M NaCl, once with LiCl, twice with Buffer B2 and once with TE for 15 minutes incubation each followed by 2 minutes centrifugation at 500g, 4°C. Captured proteins were eluted by adding 30 μL 1x BXT buffer (IBA-Lifesciences) for 30 minutes at 25°C and strong shaking (1400 rpm) and boiled for 5 minutes at 95°C. Then, the supernatant was transferred to a new tube, LSB buffer added to 1x final concentration and proteins analyzed by Western Blotting.

### Quantification and statistical analysis

#### ChIP-Seq

##### Data processing and analysis

Sequencing reads were adaptor-trimmed using TrimGalore (v0.0.6, Babraham Bioinformatics) and aligned to the mm10 mouse reference genome, the hg38 human reference genome, or the dm6 drosophila genome (6.27.1) using bowtie2 (v2.4.2^69^). Only uniquely mapping reads were kept using samtools (v1.12^70^) and duplicates removed using picard MarkDuplicates (v2.26.10, Broad Institute). Read numbers of all datasets are listed in Table S2. The generated data sets were used to define peak regions using MACS2 (v2.2.6^71^) with parameters --nomodel, read extension by model option (extension by 250 bp), using the pooled Inputs as control file and an FDR value of q = 0.05 with the broad option -b and a cutoff of q=0.1. External datasets were processed identically to own generated datasets. In case external datasets were sequenced paired-end, the paired-end function of MACS2 (v2.1.1) was used (FDR value of q= 0.05) using broad option -b and a cutoff of q=0.1.

External datasets:

- RING1B, SUZ12: Peak set (BED-file) taken from GSE132752^68^
- PCGF1 (GSE132752: GSM3891362-GSM3891364, Inputs: GSM3891380-GSM3891382)
- PCGF2 (GSE132752: GSM3891368-GSM3891370, Inputs: GSM3891380-GSM3891382)
- PCGF6 (GSE132752: GSM3891374-GSM3891376, Inputs: GSM3891380-GSM3891382)
- H3K4me3 (ENCODE project [https://doi.org/10.17989/ENCSR000CGO], datasets: ENCFF997CAQ, ENCFF425ZMW)
- RNAP-Ser5p (GSE128643: GSM3681611-GSM3681612^27^)
- CpG Islands: Peak set (BED-file) taken from GSE21442^67^ and converted to mm10 using UCSC LiftOver
- Enhancers: Enhancer set (BED-file) taken from the FANTOM consortium et al.^66^

To obtain the final peak dataset, all called peaks of the individual replicates (n= 2-3) were concatenated, sorted using the BEDtools (v2.30.0,^73^) SortBed function and overlapping peaks within 500 bp distance merged by the BEDtools Merge function. From this dataset only peaks that overlap the peak set of all individual replicates were kept using the BEDtools Intersect Interval function with -a specified and overlapping blacklisted regions were excluded. The resulting peak sets were used for subsequent analysis. H2BK120ub1-decorated genes were defined by first filtering the Ensembl gene list of mm10 (n=32,025) for not overlapping blacklisted regions and a gene length over 3000 bp to exclude short pseudogenes (n=20,380). 8,103 genes had at least one H2BK120ub1 peak in their transcribed region and 2,332 genes thereof were longer than 50kB. For HCT116 data, the Ensembl list of hg38 was used with the same filtering parameters resulting in a list of n=11,120 (>3kB, H2BK120ub1-positive), n=4,337 (>50kB, H2BK120ub1-positive) genes.

#### ChOR-Seq

##### Data processing and normalization

Sequencing reads were adaptor-trimmed using TrimGalore (v0.0.6, Babraham Bioinformatics) and aligned to the mm10 mouse reference genome, the hg38 human reference genome or the dm6 drosophila genome (6.27.1) using bowtie2 (v2.4.2^69^). Only uniquely mapping reads were kept using samtools (v1.12^70^) and duplicates removed using picard MarkDuplicates (v2.26.10, Broad Institute). Read numbers of all datasets are listed in Table S2.

For RRPM-based analysis, bam files were imported into Seqmonk (v1.47.1) using standard parameters, ignoring duplicates and extending reads by 250 bp. To account for spike-In normalization, samples were normalized as described in Fursova et al.^53^ Using the EdU-purified Input samples (“ClickedInputs”) as reference for relative spikeIn abundance and EdU labelling efficiency, the downsampling factor was calculated as follows (uniquely mapping, deduplicated reads in millions):

$$Downsampling\;factor=\alpha *\frac{1}{ChOR\;dm6\;reads}*\;\frac{ClickedInput\;\left(dm6\;reads\right)}{ClickedInput\;\left(mm10\;or\;hg38\;reads\right)}$$

For a given time course, the largest downsampling factor was set to 1 by the α coefficient and all other factors adjusted accordingly. The individual downsampling factor was then applied to imported datasets in Seqmonk.

For visualization, the RRPM-normalized datasets were merged, exported as bedgraph in 50 bp intervals and visualized across selected regions with Seqplots (v1.12.1^74^). When indicated, the signal was log_2_ or z-score ($z=\frac{x-\mu }{\sigma }$) normalized to focus on distribution differences (μ = mean of population, σ = standard deviation).

To visualize global restoration curves (Figure 4G), the downsampling factor (without the α coefficient) was multiplied by the number of uniquely mapped, deduplicated mm10 reads of the ChOR sample (in million reads) and plotted in replicates using R (v4.1.2) in RStudio (v2021.9.2.382). The restoration regression equation follows the calculations of a first order equation^48^:

$$y=y\left(\max\right)-b*e^{\left(-k*t\right)}$$

*y(max)* = maximum (saturated) level of the evaluated histone mark/variant

t=time

k=kineticparameter

*b* = mark-specific parameter, reflecting the relative increase from y(t=0) to y(max)

Following this equation, the initial amount at T0 can be calculated as *y(t=0)=y(max)–b*. Accordingly, the time needed to restore 90% of the final signal *y(t)=0.9^∗^y(max)* can be calculated as *t=ln(0.1^∗^ y(max)/b)/-k*.

In our analysis, the T480 time point for H2AK119ub1 and H2BK120ub1 as well as the T180 timepoint for H2A.Z was excluded to ensure accurate regression analysis. Indeed, both H2AK119ub1 and H2BK120ub1 are reported to be removed during mitosis, potentially resulting in varying levels at T480 which reflects cell cycle stage G1.^81^ Likewise, H2A.Z was reported to be removed from promoters prior to mitosis, potentially explaining the discrepancy of the T180 timepoint.^82^

For RPM-based analysis of genome occupancy, bam files were converted to bigwig files using deepTools (v3.5.1^72^) bamCoverage using standard parameters with options --ignoreDuplicates --bl Ensembl mm10 blacklist --e 250 and normalized to counts per million (RPM). Pearson correlation plots of replicates were generated using multiBigwigSummary and plotCorrelation from deepTools with options –skipZero and –removeOutliers. For heatmaps and peak profiles, replicates were merged and plotted using deepTools computeMatrix and plotHeatmap with --missingDataAsZero specified. To visualize genome occupancy, bam files were imported into Seqmonk, read normalized to reads per million (RPM), replicates merged and visualized using Seqmonk’s visualization tool (window size 5kB (H2A.Z: 2.5kB), step size 1kB).

##### Restoration category analysis

RRPM-normalized datasets were summed across entire peaks (normalized to peak width) or peaks parsed into specified window sizes (normalized to window size: 1kB for H2AK119ub1, H2A.Z and H3K27me3; 2kB for H2BK120ub1) using Seqmonk. Subsequently, data sets were analysed in R following the restoration analysis as described in Reverón-Gómez et al.^16^ In brief, a peak was considered restored at time point R(X) when the signal did not increase more than 1.5-fold in subsequent time points T(X+n) (i.e. T(X+n)/T(X) < 1.5). Only peaks were considered that restored at the same time or category in both replicates. For nascent H2AK119ub1 impacting restoration kinetics, nascent H2AK119ub1 signals were stratified into deciles according to intensity (D1, D2, …, D10) and the high (D1), medium (D5), and low (D10) groups were analysed for restoration categories.

Peaks with absent values over the entirety of the time course were excluded and peaks with decreasing values (where the signal decrease 1.5-fold) were classified as unstable peaks. Analysis and Filtering of restoration signal across different genomic features was done in R using Bioconductor and related packages.^75^

##### Gene expression categorisation

RNA-Seq data from three replicates in mES cells was quantified in Seqmonk using the integrated RNA-Seq analysis pipeline (options: merged transcripts, paired end, length corrected, DNA contamination corrected, log transformed, strand specific), averaged, and divided into four quartiles according to their expression values. If appropriate, the lowest two quartiles (0-25%, 25-50%) were merged for analysis of active chromatin marks or excluded from analysis. For correlation with gene expression, only genes that contain peaks of the interrogated marks in their promoter region (within 2 kB from the respective TSS) were considered. Peaks overlapping multiple promoters were excluded from analysis. For H2BK120ub1 correlation with gene expression, the parsed peaks in 2kB windows were filtered for overlap with promoter regions (+/- 1 kB).

#### SCAR-Seq

##### Data processing & normalization

Reads were processed and mapped to the mm10 mouse reference genome as described above. Read numbers of all datasets are listed in Table S2. The resulting processed bam files were split into forward and reverse strands according to the SAM flag, using samtools view (version 1.5) -F 20 and -f 16, respectively.

External datasets:

Okazaki-Seq: GSE117274 (GSM3290342)^23^

Histone partition signal was computed as in Petryk et al.^23^ For each strand the SCAR normalized signal (RPM) was computed in 1 kb windows and smoothed in a uniform blur considering the neighbouring 30 bins on each side. For each 1 kb window, the signal from its corresponding SCAR input was subtracted and negative values were set to zero. Input corrected windows with RPM < 0.3 on both strands were filtered out and not considered for further analyses. The final partition score for each 1 kb window was calculated as:

Partition=(F-R)/(F+R)

where F and R correspond to the number of normalized and input-corrected reads for the forward and reverse strand, respectively. The partition value relates to the ratio of histones with a specific modification being segregated to the nascent forward (Partition > 0) or nascent reverse (Partition < 0) strand within each window respectively. Extreme partition ratios (values > 0.9999 quantile or < 0.0001 quantile) were set to the quantile value.

Okazaki-Seq replication fork directionality (RFD) scores and filtered initiation zones (IZs) for mESC were taken from Petryk et al.^23^ and used as focus points to define replication via leading or lagging strand mechanism. The RFD score in Okazaki-Seq is calculated like SCAR-Seq partition scores but subtracting the forward (F) strand signal from the reverse (R) strand signal instead:

RFD=(R-F)/(F+R)

##### Data analysis

For each mark (H3K27me3, H2AK119ub1, H4K20me0, H2A.Z and JARID2) only IZs zones within 100 kb of WT ChIP-Seq defined peaks were used for further analysis. Initiation zone edges, where the RFD reach local extrema, were determined within 100 kb upstream and downstream of the initiation zone, by selecting the location with minimum and maximum RFD value, respectively. A window size of 200 kb around each initiation zone was chosen based on the average initiation zone size (from upstream to downstream initiation zone edge) (mean size = 112 kb) and the distances to neighbouring initiation zones (mean distance = 359 kb). This left a total of n = 2076, 695, 1143, 1647, 1163 IZs used for downstream analyses for H3K27me3, H2AK119ub1, H4K20me0, H2A.Z and JARID2, respectively. The average partition signal from all replicates was used for visualization purposes in Partition line plots (Figures 3 and 7). The Partition line plot for H4K20me0 was plotted across the IZs of H2AK119ub1 for simplicity.

The difference in SCAR-Seq partition ratios at initiation zone edges between leading and lagging enriched strands were tested with a Wilcoxon signed-rank test. The test was performed between each pair of SCAR-Seq samples (wild type or mutant, different time points), taking all 1 kb windows with sufficient coverage (RPM > 0.6), between 25 and 75 kb from its nearest initiation zone. To compare partition values on both sides of the initiation zones, an adjusted partition score was computed by negating the partition values of all windows upstream of the initiation zone. If co-overlapping features were evaluated, windows were further filtered for the presence/absence of features. The resulting asymmetry boxplots are shown in Figures 7, S3, and S7.

## Data Availability

• ChIP/ChOR/SCAR-Seq data have been deposited at GEO under GSE204988 and are publicly available as of the date of publication. Accession numbers are also listed in the key resources table. Original western blot images have been deposited at Mendeley under [https://doi.org/10.17632/7pxmj6dnp9.1] and are publicly available as of the date of publication. The DOI is also listed in the key resources table. • This study did not generate original code. All computational approaches and software used are described in the STAR Methods and listed in the key resources table. • Any additional information required to reanalyse the data reported in this work is available from the lead contact upon request.

## Supplemental information

Table S1. Oligonucleotides and targeting constructs used in this study, related to STAR Methods

Table S2. Sequencing data generated in this study, related to STAR Methods
