# Supplementary material for: Recycling of modified H2A-H2B provides short-term memory of chromatin states
Source: Cell. 2023 Mar 2;186(5):1050–1065.e19. doi: 10.1016/j.cell.2023.01.007 (PMC9994263; doi:10.1016/j.cell.2023.01.007)
Supplement: Table S1. Oligonucleotides and targeting constructs used in this study, related to STAR Methods [file mmc1.pdf]

Cell, Volume *186*

Supplemental information

Recycling of modified H2A-H2B provides short-term memory of chromatin states

Valentin Flury, Nazaret Reverón-Gómez, Nicolas Alcaraz, Kathleen R. Stewart-Morgan, Alice Wenger, Robert J. Klose, and Anja Groth

Supplementary Table S1: Oligonucleotides and targeting constructs used in this study. Related to STAR methods.

Name	Use	Sequence
Talen-F-Pola1	Pola1-3A generation	HD NI NN NN NI NG NN NI NG NN NI HD NG NN NN NI NG NG NN NG (Targeting CAGGATGATGACTGGATTGT)
Talen-R-Pola1	Pola1-3A generation	NN HD HD NG NN NN HD NI NN NN HD NI NI NG NG NG HD HD NG (Targeting GCCTGGCAGGCAATTTCCT)
Oligonucleotide donor*	Pola1-3A generation	GAAGAGCAGTATTCGAAACTGGTTCAGGCCCGTCAGGATGATG**C**C**GC**GATTGT***C***GA***C***G**C**CGGTATGTGGGAGGAAATTGCCTGCCAGGCACTGTGCTACTTC
pRR_Puro/eGFP reporter sequence	Pola1-3A generation	CAGGATGATGACTGGATTGTGGATGACGGTATGTGGGAGGAAATTGCCTGCCAGGC
Colony-F	Pola1-3A generation	GCAGGTTGAGGACCTCACAAGTG
Colony-R	Pola1-3A generation	GAAAATGTCTCCTCAGGCATAGCAAC
Talen-F-MCM2	MCM2-2A generation	HD NG HD NG NG NN HD NI NN NI NN NI HD NG NI HD HD NN NG (targets: CTCTTGCAGAGACTACCGT)
Talen-R-MCM2	MCM2-2A generation	HD HD HD NG HD NN NN HD HD NG HD NN NG NI NN NI HD NI NG (targets: CCCTCGGCCTCGTAGACAT)
Oligonucleotide donor**	MCM2-2A generation	TTTGGGGATTCATTGTCCACTGTTGGTCTCTTGCAGAGAC**GC**CCGTCCCATTCCGGAGCTCGATGTC**GC**CGAGGCCGAGGGATTGGCCCTGGATGATGAAGATGTGGAG
pRR_Puro/eGFP reporter sequence	MCM2-2A generation	CTCTTGCAGAGACTACCGTCCCATTCCGGAGCTCGATGTCTACGAGGCCGAGGG
Colony-F	MCM2-2A generation	ATCTAGAGGAAGCACTGGCCAC
Colony-R	MCM2-2A generation	GAAGTTCTTGAAGCGGTGGTGG
sgRNA#1	BAP1-dTAG generation	CACTGGCGTTTGGCCTTGTA
Colony-F	BAP1-dTAG generation	TGTGAGAGCCTGGGAGACAG
Colony-R	BAP1-dTAG generation	CAAGAGAGGCTGCAGAGTCAG
sgRNA#1	POLE4KO generation	CACGTTTCGGGAGGGGATGG
sgRNA#2	POLE4KO generation	CTCTACCCAAATCTCTCCTC
Colony-F	POLE4KO generation	AAGGGGCCGAAATCGCG
Colony-R	POLE4KO generation	TCCCCTTGCTTCAATGATGCC
Colony-R2 (deleted region)	POLE4KO generation	GCAATCCTGTGTAGACGTGGAC

* Six mutations to introduce D83A, W84A and D88A are underlined. The silent mutations V86V and D87D create a *SalI* restriction site and are represented in italics.

** Four mutations to introduce Y81A and Y90A are underlined. The mutations disrupt the *AccI* restriction site, which was used as a readout for genotyping
